# Cardiometabolic protein expression levels and pathways associated with kidney function decline in older European adults with advanced kidney disease

**DOI:** 10.1093/ckj/sfaf079

**Published:** 2025-03-18

**Authors:** Ryan E Aylward, Samantha Hayward, Nicholas C Chesnaye, Roemer J Janse, P Andreas Jonsson, Claudia Torino, Antonio Demetrio Vilasi, Maciej Szymczak, Christiane Drechsler, Friedo W Dekker, Marie Evans, Kitty J Jager, Christoph Wanner, Brian Rayner, Yoav Ben-Shlomo, Nicki Tiffin, Fergus J Caskey, Kate Birnie

**Affiliations:** Population Health Sciences, Bristol Medical School, University of Bristol, Bristol, UK; Division of Nephrology and Hypertension, Faculty of Health Sciences, University of Cape Town, Cape Town, South Africa; Translational Health Sciences, Bristol Medical School, University of Bristol, Bristol, UK; Renal Service Southmead Hospital, North Bristol NHS Trust, Bristol, UK; ERA Registry, Department of Medical Informatics, Amsterdam UMC, University of Amsterdam, Amsterdam, The Netherlands; Amsterdam Public Health Research Institute, Quality of Care, Amsterdam, The Netherlands; Department of Clinical Epidemiology, Leiden University Medical Center, Leiden, The Netherlands; Department of Public Health and Clinical Medicine, Umeå university, Umeå, Sweden; Institute of Clinical Physiology, National Research Council, Reggio Calabria, Italy; Institute of Clinical Physiology, National Research Council, Reggio Calabria, Italy; Clinical Department of Nephrology, Transplantation Medicine and Internal Diseases, Wroclaw Medical University, Wroclaw, Poland; Division of Nephrology, University Hospital of Wurzburg, Wurzburg, Germany; Department of Clinical Epidemiology, Leiden University Medical Center, Leiden, The Netherlands; Department of Clinical Sciences Intervention and Technology, Karolinska Institutet, Stockholm, Sweden; ERA Registry, Department of Medical Informatics, Amsterdam UMC, University of Amsterdam, Amsterdam, The Netherlands; Amsterdam Public Health Research Institute, Quality of Care, Amsterdam, The Netherlands; Division of Nephrology, University Hospital of Wurzburg, Wurzburg, Germany; Division of Nephrology and Hypertension, Faculty of Health Sciences, University of Cape Town, Cape Town, South Africa; Population Health Sciences, Bristol Medical School, University of Bristol, Bristol, UK; South African Medical Research Council Bioinformatics Unit, University of Western Cape, Cape Town, South Africa; Population Health Sciences, Bristol Medical School, University of Bristol, Bristol, UK; Renal Service Southmead Hospital, North Bristol NHS Trust, Bristol, UK; Population Health Sciences, Bristol Medical School, University of Bristol, Bristol, UK

**Keywords:** chronic kidney disease, fibrosis, progression, proteins

## Abstract

**Background:**

Cardiovascular disease and chronic kidney disease (CKD) progression pathophysiology are similar. We investigated associations of cardiometabolic protein expression and pathways with kidney function decline in older adults with advanced CKD referred for nephrology assessment.

**Methods:**

Two plasma proteomic panels analysed at baseline (Olink^®^ cardiometabolic T96 and cardiovascular II T96, Uppsala, Sweden) and longitudinal estimated glomerular filtration rate (eGFR) data from European adults aged >65 years with a single eGFR of <20 mL/min/1.73 m^2^ [European Quality (EQUAL) Study] were used to explore mechanisms of CKD progression. Protein-slope associations were estimated using generalized linear mixed-effects models and with a false-discovery rate *P* < .05 taken to validation to verify the effect size of the association. Proteins were further modularized into biological pathways using pathway enrichment analysis.

**Results:**

A discovery sub-cohort of 238 complete-case participants from Germany, the UK and Poland (median age 76 years, 41% female sex, median baseline eGFR 17.8 mL/min/1.73 m^2^) were included and 246 participants from Sweden formed the validation sub-cohort (median age 75 years, 28% female, median baseline eGFR 17.5 mL/min/1.73 m^2^). Of the 175 analysed proteins, higher expression levels of Receptor-type tyrosine-protein phosphatase S [–15.4% change in eGFR per year per doubling of protein expression; 95% confidence interval (CI) –23.5%, –7.6%], Insulin-like growth factor binding protein 6 (–7.9%; 95% CI –12.3%, –3.5%) and Ficolin 2 (–7.4%; 95% CI –12.0%, –2.8%) showed a validated association with eGFR decline.

**Conclusions:**

Higher expression levels of proteins and biological pathways involving fibrogenesis and the complement cascade were found to be associated with kidney function loss. However, study limitations and unavailability of concurrent kidney cellular proteomic signatures necessitate further study.

## Graphical Abstract

**Graphical Abstract:**
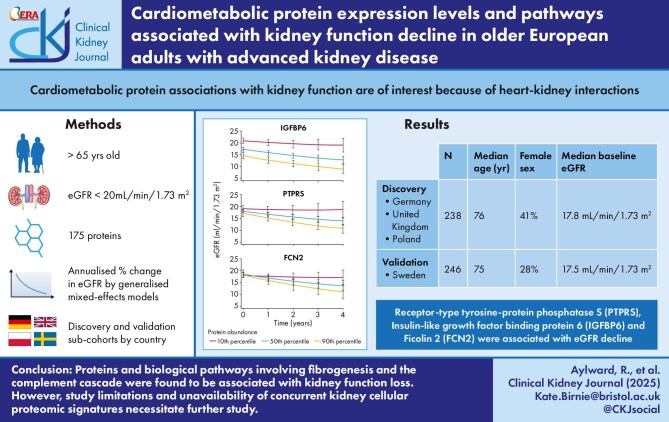

KEY LEARNING POINTS **What was known:**

- Pathophysiological mechanisms that drive ischaemic heart disease, atherosclerosis and heart failure share traditional risk factors as well as common biological pathways such as inflammation, oxidative stress and thrombosis which are responsible for chronic kidney disease (CKD) progression.

**This study adds:**

- In advanced CKD, higher expression levels of proteins that are involved in extracellular matrix organization, fibrogenesis and complement cascade pathways were associated with kidney function loss in older European adults.

**Potential impact:**

- These dysregulated pathways may be the final common pathway leading to kidney damage and key molecules operating in these pathways may be interrogated to identify biomarkers for impending fibrosis or therapeutic targets to ameliorate fibrosis.

## INTRODUCTION

Risk factors for the development and progression of kidney and cardiovascular disease share many similarities, suggesting that cardiovascular disease proteins and biological pathways are important mechanisms of kidney function loss [1]. A Chronic Renal Insufficiency Cohort (CRIC) sub-study observed an association between myocardial stretch, inflammatory and remodelling proteins and chronic kidney disease (CKD) progression, highlighting potential shared cardio-renal biological pathways [2].

A previous study assayed cardiovascular and inflammation multiplex proteomic panels, developed by Olink^®^ (www.olink.com, Uppsala, Sweden). A discovery cohort was derived from the Prospective Investigation of the Vasculature in Uppsala Seniors Study (PIVUS) and replicated in the Uppsala Longitudinal Study of Adult Men (ULSAM); none had advanced kidney dysfunction at the time of inclusion [estimated glomerular filtration rate (eGFR) <30 mL/min/1.73 m^2^] [3]. Circulating proteins involved in phosphate homeostasis, inflammation, apoptosis, extracellular remodelling, angiogenesis and endothelial dysfunction were found to be associated with kidney function decline. However, these findings were limited to sex- and region-restricted study populations, and participants with advanced CKD were not represented.

Currently available therapeutics to control traditional cardiovascular risk factors are limited as CKD progression is only modestly ameliorated especially in persons with established advanced CKD [4, 5]. It is therefore important to discover new and reaffirm canonical evidence of specific biological pathways that mechanistically explain drivers of progression that may be therapeutically targetable. This study aims to contribute to the knowledge gap by investigating the slope of eGFR decline relative to cardiovascular protein levels collected at baseline and the pathways these proteins represent in older Europeans with advanced age and CKD.

## MATERIALS AND METHODS

### Study population

Participants of the European Quality (EQUAL) Study, who additionally consented to proteomic analysis, were included in this sub-study [6, 7]. In the EQUAL Study, participants aged ≥65 years referred to participating nephrology centres in Germany (DE), the UK, Poland (PL), Sweden (SE), Italy (IT) and the Netherlands (NL) with an eGFR <20 mL/min/1.73 m^2^ (calculated by Modification of Diet in Renal Disease equation, without race coefficient) were followed up over time. Recruitment was from 2012 through 2018. Follow-up continued until death, loss to follow-up, study withdrawal, kidney replacement therapy (KRT) initiation or end of study at 4 years after entry. Ethical institutional approvals in participating countries were obtained prior to commencement of the study and conducted in accordance with the declaration of Helsinki. Written informed consent was provided for all participants included in the EQUAL Study to collect clinical information and, separately, bio-samples.

### Exposure

#### Analysis of individual proteins

Two proteomic panels, cardiometabolic T96 and cardiovascular II T96, offered by Olink^®^ laboratories, Uppsala, Sweden were assayed on plasma samples taken at the baseline study visit (www.olink.com); see Supplementary data, methods S1.1 and S1.2. Only data from participants from DE, the UK, PL and SE were available. Participant samples from DE, the UK and PL were batched together during proteomic analysis and approximated the number of samples from SE. Therefore, the former countries were used as the discovery sub-cohort and Swedish participants as validation of the effect size of the associations.

#### Analysis of regulatory pathways

Reactome (reactome.org) is a database that catalogues the known relationships between proteins and genes in regulatory and functional pathways in the cell. The *Homo sapiens* catalogue was queried using universal protein accession numbers [8]. Pathway enrichment analysis was performed to determine whether particular pathways were overrepresented in the analysed Olink^®^ panels more than by chance alone; see Supplementary data, methods section 1.3 [9]. Principal component analysis was then used to modularize groups of individual proteins, that were not overrepresented, belonging to the same biological pathway into fewer principal component scores rather than individual protein abundance levels for analysis relative to eGFR slope.

#### Outcome

The outcome of interest was in estimating the eGFR slope by using repeated measures of pre-KRT eGFR values. The Chronic Kidney Disease Epidemiology Collaboration (CKD-EPI) 2009 equation was used to estimate GFR from baseline to end of follow-up at 6-monthly intervals (or 3-month intervals when eGFR reached <10 mL/min/1.73 m^2^) according to the EQUAL Study protocol [7].

#### Mitigation of bias

Five sequential model adjustments were performed: unadjusted Model 0, Model 0 + age (continuous variable), sex and country (demographic Model 1), Model 1 + systolic blood pressure (SBP), diabetes mellitus status, and primary renal disease (clinical Model 2), Model 2 + albuminuria [albumin:creatinine ratio (ACR) Model 3], Model 3 + prescribed medications (medications Model 4) (see Supplementary data, Fig. S2). SBP was measured using a standardized operating procedure. Diabetes mellitus status, medications and primary renal disease, as defined by the European Renal Association, were transcribed from medical records [10]. Albuminuria was quantified as a spot or 24-h urine collection ACR. All laboratory testing was performed as part of usual care at the local study site. Medications included any use of renin–angiotensin–aldosterone system inhibitors (angiotensin-converting enzyme inhibitors, aldosterone receptor blockers and mineralocorticoid receptor antagonists) and β-blockers at the time of the baseline visit. Model 3 was of primary interest as it was not expected that medications would affect all protein levels; Model 4 was of secondary interest.

### Analysis

#### Primary analysis

A complete case analysis was undertaken as there was little evidence that the outcome influenced the chance of being a complete case or not (see Supplementary data, results section 2.2 for details) [11]. Generalized linear multilevel mixed-effects models were used to analyse the repeated measures of eGFR. Random intercepts and slopes were included to allow for the variation within and between individuals to be captured in the overall estimate. A Poisson distribution with log-link was used, as recommended in place of logarithmic transformation of the outcome (the distribution of eGFR was not normally distributed) [12]. Robust standard errors relaxed the assumption that the variance must equal the mean in Poisson regression analysis [13, 14]. The multiplicative interactions between protein and time were examined to see how proteins were related to eGFR slope, and coefficients were reported as annualized percentage change [13]. To visualize the slope estimates produced by Model 3, predictions of eGFR on the original mL/min/1.73 m^2^ scale at fixed time points (yearly) and percentiles (10th, 50th and 90th) of protein abundance were graphed. Baseline eGFR was included in the repeated measures eGFR and not adjusted for separately. Given multiple testing, protein-slope associations with a Benjamini–Hochberg false-discovery rate *P*_FDR_-value <.05 were taken to validation to confirm that the effect estimates were sustained and a nominal *P*-value <.05 was taken as successful validation [15].

#### Sensitivity analyses

Two sensitivity analyses were conducted. Firstly, eGFR values become missing after events such as KRT initiation, death and loss to follow-up. eGFR slope may be a direct cause of these occurrences, so censoring becomes informative [16]. A joint model, that simultaneously models the longitudinal eGFR slope and the time to occurrence of the above competing events, was used to investigate whether protein-slope associations changed in the presence of informative censoring (see Supplementary data, methods section 2.1) [17]. Secondly, although a complete-case analysis was deemed unlikely to be biased (Supplementary data, methods section 1.4.), multiple imputation of missing ACR values was used to determine whether the protein-slope estimates were comparable to those estimated in the primary analysis (Supplementary data, section 1.5.). Ten-donor draw predictive mean matching was used in which the imputed value is derived from a subset of observed ACR values of similar predictive mean as recommended in the case of non-normally distributed data [18]. All variables in the substantive model (the analysis of interest), including the exposure, independent variables, outcome (repeated measure eGFR) and the protein*time interaction, were included in the imputation model. Rubin's rules were used to combine the imputation model estimates of 50 imputed datasets [19].

## RESULTS

### Patient characteristics

Overall, a complete case analysis was undertaken on 484 individuals, approximately equally split between discovery and validation sub-cohorts (Table 1 and Supplementary data, Fig. S4). Baseline characteristics are shown in Table 1. A total of 4472 pre-KRT eGFR values over time were available to estimate eGFR slope. Although an eGFR of <20 mL/min/1.73 m^2^ in the past 6 months was mandatory for eligibility, a small number of individuals had eGFR values closer to 60 mL/min/1.73 m^2^ at baseline, and were nevertheless included (Supplementary data, Fig. S5).

**Table 1:** Baseline characteristics of participants under study.

	Overall cohort, *N* = 484	Discovery cohort, *n* = 238	Validation cohort, *n* = 246
Age in years, median (IQR)	75 (70; 81)	76 (69; 81)	75 (70; 80)
Female sex, %	34	41	28
Country, %			
Germany	9.2	18	0
UK	30	59	0
Poland	12	23	0
Sweden	49	0	100
Comorbidities, yes %			
Diabetes mellitus	40	43	37
Hypertension	88	84	91
Coronary artery disease	26	28	23
Heart failure	19	17	21
Primary renal disease, %			
Glomerular	11	8.8	13
Tubulo-interstitial	10	11	9.3
Diabetes	23	24	22
Renovascular	35	30	41
Other systemic disease	3.6	3.6	3.7
Hereditary	4.4	3.2	5.7
Miscellaneous	13	19	6.1
Clinical measurements			
Current smoking, yes %	7.0	6.9	7.0
SBP mmHg, median (IQR)	146 (131, 160)	145 (130, 160)	148 (131, 160)
BMI kg/m^2^, median (IQR)	27.7 (24.5, 31.2)	28.3 (24.7, 32.1)	27.0 (24.4, 30.2)
*N* of eGFR values per person, median (IQR)	5 (2, 8)	4 (2, 6)	7 (3, 9)
Laboratory measurements, median (IQR)			
eGFR CKD-EPI, mL/min/1.73 m^2^^a^	17.7 (14.6, 20.8)	17.8 (14.3, 21.1)	17.5 (15.1, 20.5)
eGFR slope, mL/min/1.73 m^2^^a^ per year (95% CI)	–1.96 (–2.24, –1.68)	–1.44 (–1.92, –0.96)	–2.29 (–2.64, –1.94)
eGFR slope, % change per year (95% CI)	–14.6 (–16.7, –12.5)	–11.0 (–14.0, –7.9)	–16.5 (–19.4, –13.6)
ACR, mg/mmol	42 (8, 173)	44 (6, 174)	39 (10, 169)
Calcium, mmol/L	2.27 (2.17, 2.37)	2.27 (2.15, 2.37)	2.27 (2.19, 2.38)
Phosphate, mmol/L	1.29 (1.12, 1.48)	1.27 (1.13, 1.44)	1.30 (1.11, 1.50)
Parathyroid hormone, pmol/L	16 (10, 23)	16 (11, 25)	15 (9, 22)
Total cholesterol, mmol/L	4.60 (3.80, 5.50)	4.59 (3.90, 5.50)	4.60 (3.80, 5.50)
KFRE, median (IQR)^b^			
4-variable, 2-year	15.3 (5.8, 32.9)	15.3 (7.3, 31.7)	15.7 (4.6, 34.9)
8-variable, 2-year	18.5 (7.2, 34.3)	18.5 (8.1, 33.0)	18.3 (6.4, 37.1)
4-variable, 5-year	47.5 (20.6, 78.7)	47.4 (25.4, 77.1)	48.3 (16.6, 81.0)
8-variable, 5-year	60.1 (28.4, 84.9)	60.1 (31.6, 83.5)	59.8 (26.3, 87.6)

a The annualized eGFR slopes were calculated without proteins and adjusted for age, sex, diabetes status, SBP, primary renal diagnosis, country and ACR using generalized linear mixed effects modelling.

b The Kidney Failure Risk Equation (KFRE) predicts the risk of developing kidney failure within 2 and 5 years using the four predictors age, sex, eGFR and log(ACR), or eight predictors age, sex, eGFR, log(ACR), serum calcium, serum phosphate, serum bicarbonate and serum albumin [46].

IQR, interquartile range; BMI, body mass index.

### Primary analysis of individual protein–kidney function associations

Of 184 proteins, 11 proteins did not reach assay level of detection: liver carboxylesterase 1, neutrophil defensin 1, prolyl endopeptidase, integrin alpha-M, neutrophil gelatinase-associated lipocalin, latent-transforming growth factor beta-binding protein 2, platelet-activating factor acetyl hydrolase, superoxide dismutase 1 and uromodulin. As uromodulin and superoxide dismutase 1 are expected to decrease in CKD, low levels may have a plausible association with kidney function decline and were not removed from the analysis. Therefore, the total number of proteins for analysis was 175 [20, 21].

**Figure 1:**
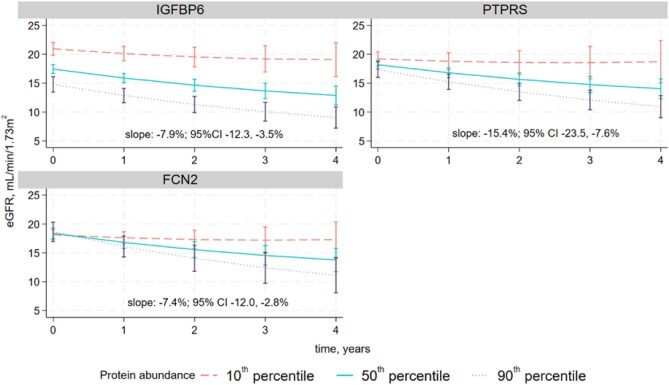
eGFR slope for each successfully validated protein in the primary analysis. Horizontal lines represent the slope and vertical bars show the 95% confidence limits. Note that the effect of GFR change is per protein concentration doubling as 1 NPX change in protein is relative and uninterpretable. Estimates are for Model 3 (discovery sub-cohort) although only IGFBP6 and PTPRS were successfully validated in that adjusted analysis (FCN2 was only validated in Model 4). Time was truncated to 4 years because of a questionable upstroke in the predicted eGFR slope after this time, likely due to sparse data >4 years (see Supplementary data, Fig. S4). Slope estimates % change per year per doubling in protein concentration are included as inset text.

**Figure 2:**
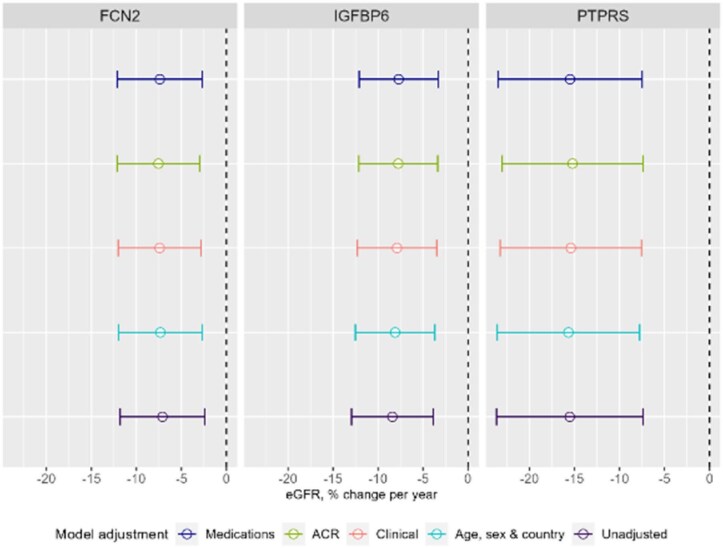
Comparison of the protein-slope estimates computed after sequential model adjustments. Hollow circles represent the slope estimates and the horizontal bars represent the 95% CIs. Note that the effect of GFR change is per protein concentration doubling as 1 NPX change in protein is relative and uninterpretable. CIs do not cross the null (0%) which would suggest an association but the CIs could not be adjusted for Benjamini–Hochberg multiple testing correction unlike the *P*-values, hence the discrepancy that the CI for FCN2 does cross the null for the medications-adjusted model (Model 4) but was still considered as having no evidence of an association with eGFR slope for this model adjustment. Demographic adjustment (Model 1): age, sex and country. Clinical adjustment (Model 2): Model 1 + primary renal disease, diabetes mellitus status and SBP. ACR adjustment (Model 3): Model 2 + ACR. Medications (Model 4): Model 3 + renin–angiotensin–aldosterone system inhibitors and β-blockers.

In the discovery sub-cohort, 78 proteins were positively associated with eGFR slope, but none had evidence of an association using the threshold of *P*_FDR_ < .05 (Supplementary data, Fig. S6). Of the 97 proteins showing a negative association with eGFR slope, 5 were taken to validation: Receptor-type tyrosine-protein phosphatase S (PTPRS), Insulin-like growth factor binding protein 6 (IGFBP6), Transforming growth factor beta receptor 3 (TGFBR3), ficolin-2 (FCN2) and Procollagen C-endopeptidase enhancer 1 (PCOLCE). Higher levels of PTPRS were found to have the largest association with eGFR decline [–15.4% per year per protein concentration doubling; 95% confidence interval (CI) –23.5, –7.6%] which was sustained in the validation analysis (–12.4%; 95% CI –20.1, –4.7%) (see Supplementary data, Fig. S6). IGFBP6 demonstrated smaller albeit more consistent effect sizes in the discovery (–7.9%; 95% CI –12.3, –3.5%) and validation sub-cohorts (–7.4%; 95% CI –12.3, –2.6%) compared with PTPRS. The FCN2 estimates were –7.4% (95% CI –12.0, –2.8%) in discovery and –5.0% (95% CI –9.1, –1.0%) in validation. FCN2 was associated with eGFR decline but not after additional adjustment for medications. The proteins PCOLCE and TGFBR3 showed no replicable associations in the validation sub-cohort. Slope-estimates for the discovery primary adjusted analysis are shown in Supplementary data, Fig. S6 for all proteins and eGFR slopes over time are shown for the three successfully validated proteins in Fig. 1. Slope effect estimates did not differ substantially using different model adjustments (Fig. 2).

### Sensitivity analysis

Estimates of the association of proteins with eGFR decline derived using the joint model were similar compared with the primary analysis, suggesting a negligible bias introduced by informative censoring and competing risks (Supplementary data, Fig. S7). Imputation model slope estimates were comparable to the CCA, though there was evidence that additional proteins were associated with eGFR decline (see Supplementary data, results section 3.2). These were: EGF-containing fibulin-like extracellular matrix protein 1 (EFEMP1), Cluster of differentiation 59 (CD59), Cystatin 3 (CST3), Immunoglobulin lambda-2 chain C regions (IGLC2), Neural cell adhesion molecule 1 (NCAM1), Procollagen C-endopeptidase enhancer 1 (PCOLCE), Factor 7 (F7) and mast/stell cell receptor Kit (KIT). The eGFR slopes were most often less steep (eight proteins) but consistent (overlapping CIs) with the primary analysis (shown in Supplementary data, Fig. S7).

### Secondary analysis of biological pathway–kidney function associations

Analysis of 35 individual proteins that were associated with eGFR decline (meeting a liberal threshold of *P* < .05) identified 373 biological pathways in the Reactome knowledgebase. There was evidence of enrichment for 11 pathways (Table 2). Of these, five pathways have functions beyond ubiquitous intracellular signalling and were analysed further using principal component scores relative to eGFR slope. These were: extracellular matrix (ECM) organization, regulation of insulin-like growth factor (IGF) transport and uptake by IGF binding proteins (IGFBPs), phosphoinositide 3-kinase (PI3K)/protein kinase B (Akt) signalling, the complement cascade and mast/stem cell growth factor receptor kit (KIT) signalling. Supplementary data, Figs S8–S12 show the variation in principal components that individual proteins contributed to each biological pathway.

**Table 2:** Biological pathways identified using over-representation analysis.

Pathway name	Proteins found	Total^a^	FDR *P*-value
Regulation of IGF transport and uptake by IGFBPs	6	124	.001
Post-translational protein phosphorylation	5	107	.005
ECM organization	7	300	.006
Transport of gamma-carboxylated protein precursors from the endoplasmic reticulum to the Golgi apparatus	2	9	.031
Removal of amino-terminal pro-peptides from gamma-carboxylated proteins	2	10	.031
Gamma-carboxylation of protein precursors	2	10	.031
PI5P, PP2A and IER3 regulate PI3K/Akt signalling	4	118	.031
Gamma-carboxylation, transport and amino-terminal cleavage of proteins	2	11	.031
Negative regulation of the PI3K/Akt network	4	125	.031
Complement cascade	4	146	.049
Regulation of KIT signalling	2	16	.049

A search for the analysed proteins was undertaken using the Reactome knowledgebase (www.reactome.org, Creative Commons BY 4.0 license).

a Proteins known to be involved in the pathway and are catalogued within reactome. PTPRS protein is involved in the ‘extracellular matrix organization’ pathway and IGFBP6 is involved in ‘Regulation of Insulin-like Growth Factor (IGF) transport and uptake by Insulin-like Growth Factor Binding Proteins (IGFBPs)’. FCN2 protein functions in the complement cascade.

FDR, false-discovery rate; PI5P, phosphatidylinositol 5-phosphate; PP2A, protein phosphatase 2; IER3, immediate early response 3.

Predicted slope estimates are shown in Table 3 for the five pathways. The net percentage change is notably lower than for the individual proteins alone and only some principal component-slope estimates show evidence of meaningful eGFR change.

**Table 3:** Biological pathway slope estimates for the first three principal components.

Pathway name	eGFR % change per year (95% CI)	
Principal component	Discovery cohort	Validation cohort
Regulation of IGF transport and uptake by binding proteins		
PC 1	–2.1 (–3.6, –0.7)	–1.2 (–2.8, 0.5)
PC 2	4.2 (1.3, 7.1)	0.5 (–1.8, 2.8)
PC 3	2.2 (–1.0, 5.4)	0.7 (–2.4, 3.8)
ECM organisation		
PC 1	–2.2 (–3.7, –0.8)	–2.5 (–4.1, –1.0)
PC 2	3.4 (0.5, 6.2)	1.4 (–1.5, 4.3)
PC 3	2.2 (–1.3, 5.8)	–0.5 (–4.0, 3.0)
PI3K/Akt signalling^a^		
PC 1	–3.7 (–7.6, 0.3)	1.4 (–3.3, 6.1)
PC 2	4.8 (0.9, 8.7)	0.2 (–5.2, 5.5)
PC 3	1.2 (–3.7, 6.2)	–5.0 (–9.5, –0.5)
Complement cascade		
PC 1	–4.6 (–8.2, –1.1)	–5.5 (–8.6, –2.4)
PC 2	1.5 (–3.3, 6.2)	–3.3 (–9.0, 2.4)
PC 3	–1.8 (–9.7, 6.1)	–4.5 (–10.8, 1.8)
KIT signalling		
PC 1	–3.1 (–6.7, 0.6)	0.5 (–3.2, 4.2)
PC 2	4.7 (0.8, 8.6)	1.0 (–4.2, 6.1)
PC 3	–1.8 (–6.1, 2.5)	5.5 (1.1, 10.0)

For each biological pathway representing more than ubiquitous intra-cellular signalling, slope estimates are shown for the first three principal components.

a The same proteins were involved in both PI3K/Akt-related pathways.

PC, principal component.

## DISCUSSION

Using clinical, laboratory and baseline proteomic data collected in the EQUAL Study, three proteins—Receptor protein-tyrosine phosphatase sigma, FCN2 and IGFBP6—were identified as having an association with eGFR decline in two independent sub-cohorts of older Europeans with advanced CKD. This analysis contributes to the current sparse human data up until now predominated by animal studies and limited literature of these proteins’ association with CKD progression in humans.

The complementary pathway enrichment analysis found that the individual proteins that had some evidence of an association with eGFR decline were enriched in several pathways. These proteins and biological pathways are further contextualized below.

### Receptor-type tyrosine-protein phosphatase S

Binding of the trans-membrane PTPRS, an extracellular protein, with its ligand facilitates tyrosine de-phosphorylation, which modulates intracellular signalling. Chondroitin sulphate and heparan sulphate proteoglycans (CSPGs and HSPGs) act as ligands [22]. PTPRS is expressed specifically in cartilage, neuronal tissue, haematopoietic stem/progenitor cells and by the vasculature [23]. There is no literature implicating PTPRS in CKD progression but it has been implicated in ulcerative colitis activity [24] and was highly expressed in small arteries of people with CKD [25].

PTPRS is involved in the extracellular matrix organization pathway, which is consistent with fibrosis as a possible driver of CKD progression. It is unclear why Thrombospondin-2, Cadherin-1 and SPARC-like protein 1, proteins similar to those found to be associated with kidney fibrosis in other literature, and Pro-fibrotic bone morphogenetic 6, Transforming growth factor β receptor type 3 and Tissue inhibitor of metalloproteinase-1 protein were not associated with kidney function loss in this analysis [26–28].

### Insulin-like growth factor binding protein 6

IGF-I and IGF-II promote the proliferation of fibroblasts, contributing to ECM expansion and kidney fibrosis [29]. IGFBP6 primarily transports IGF-II thereby directing IGF-II to its target tissues, prolonging its half-life and regulating its activity. It is noteworthy that while IGFBP3, which carries IGF-I, is the most abundant IGFBP in circulation, is expressed by proximal tubular cells [30], and has been found to increase in people with eGFR <60 mL/min/1.73 m^2^, it was not associated with eGFR decline in this study [31]. The PI3K/Akt pathway is activated by IGF-I and is in part responsible for kidney interstitial cell hyperproliferation and fibroblast activation [32].

While there is no previous evidence that IGFBP6 is deleterious to kidney function, its gene expression is upregulated in CKD [33] and its levels have been shown to decrease in adults with kidney failure requiring kidney replacement therapy post-transplantation [34]. *IGFBP6* mRNA is highly expressed in fibroblasts within renal blood vessels in the rat kidney and to a lesser extent, interstitial cells [35]. Mast/stem cell growth factor kit (KIT) signalling is relevant here as well, as mast cells release fibrogenic factors that promote fibrosis and is upregulated in a number of glomerulonephritides [36].

In support of a glomerular process, is the finding that in immortalized human and murine models, Hale *et al*. describe the podocyte as a target for IGF-II and a reduction in IGF-II or knockout of the IGF-I receptor (IGF-IR) causes podocyte death [37]. Proteinuria was incompletely recorded in the EQUAL Study, precluding any further work to test the hypothesis that there might be a role of IGFBPs in worsening glomerular proteinuria as a mechanism for CKD progression.

### Ficolin 2

FCN2 is an extracellular protein responsible for activating the lectin complement pathway by acting as a receptor for pathogen-associated molecular patterns [38]. In a Danish study of patients with systemic lupus erythematosus, a complement-mediated autoimmune disease, low FCN2 levels, stratified by its median, predicted the development of lupus nephritis [39]. In that study, high FCN1 levels predicted development of kidney failure, defined as recorded initiation of KRT in the Danish Renal Registry, but FCN2 did not. Once a patient has received a kidney transplant, fcn2 polymorphisms have been shown to increase susceptibility to delayed graft function and acute rejection post deceased donor kidney transplantation [40].

The lectin pathway, which FCN2 activates, results in the formation of C3 convertase, an enzyme that splits C3 into C3a and C3b which then effect their immune function through chemoattraction and phagocytosis [41]. Then, C3b activates C5a which recruits further inflammatory cells. In a murine C5 gene knockout model of tubulointerstitial fibrosis, fibrosis was limited in C5 deficient mice compared with wild-type [42]. FCN2 has not specifically been linked to kidney fibrosis but lower levels predicted more severe liver fibrosis in people with non-alcoholic fatty liver disease [43]. In the current study, higher levels of FCN2 were associated with eGFR decline which is inconsistent with this finding. Glomerular diseases, often complement or immune-complex related, were the primary renal disease in ∼10% of those recruited in the EQUAL Study. This would suggest an independent role (possibly through fibrosis) for FCN2 in CKD progression beyond that previously described regarding lectin pathway/primary complement-mediated glomerular disease, such as immunoglobulin A nephropathy [44]. A recent Kidney Disease: Improving Global Outcomes (KDIGO) controversies conference has explored this possibility [45].

### Strengths and limitations

Strengths of this study are that a large panel of proteins were characterized in people of multiple European nationalities with advanced CKD. Biological pathways were also analysed and shown to be associated with eGFR decline. However, there may have been other proteins or pathways that were not tested that may be important.

The absence of a control group of people with normal kidney function for comparison and sample population of mainly European descent limits the external validity of findings in other population groups. Changes in protein concentration could not be modelled serially. In addition, data for some proteins were not available and ACR was missing in a large proportion of patients. The complete case sample size was therefore reduced. Reassuringly, computed estimates were comparable to those derived by multiple imputation.

Future work could involve externally reproducing this analysis on other groups of people with CKD in terms of geographical diversification (non-European), age (only older participants were included in the EQUAL Study) and earlier CKD stages to determine the robustness of the associations in identifying a plausible hypothesis about potential biological mechanisms of kidney function loss. These biomarkers should not be seen to replace well-established markers of kidney function. Instead, they should be explored as pathophysiological adjuncts that rather explain how kidney damage occurs and what druggable targets may be investigated therapeutically.

## CONCLUSION

The higher expression of proteins and biological pathways with links to fibrosis and the complement cascade were found to be associated with kidney function loss in this analysis. Given study limitations and unavailability of direct kidney-level proteomic signatures, causal relationships could not be established. However, the need to determine whether higher expression accelerates CKD progression in older people or is a secondary consequence of shedding from their normal locations or a result of decreased kidney excretion is highlighted. There is potential to further explore the expression of these proteins as markers of kidney pathology in lieu of histological confirmation of fibrosis and immunological confirmation of complement activation as well as potential therapeutic targets to decelerate CKD progression.

## Supplementary Material

sfaf079_Supplemental_File

## Data Availability

The clinical and proteomic assay data are available by reasonable request from the EQUAL Study investigators.
