# Correction to: Cardiometabolic protein expression levels and pathways associated with kidney function decline in older European adults with advanced kidney disease

**DOI:** 10.1093/ckj/sfaf221

**Published:** 2025-07-24

This is a correction to: Ryan E Aylward, Samantha Hayward, Nicholas C Chesnaye, Roemer J Janse, P Andreas Jonsson, Claudia Torino, Antonio Demetrio Vilasi, Maciej Szymczak, Christiane Drechsler, Friedo W Dekker, Marie Evans, Kitty J Jager, Christoph Wanner, Brian Rayner, Yoav Ben-Shlomo, Nicki Tiffin, Fergus J Caskey, Kate Birnie, for the EQUAL investigators, Cardiometabolic protein expression levels and pathways associated with kidney function decline in older European adults with advanced kidney disease, *Clinical Kidney Journal*, Volume 18, Issue 4, April 2025, sfaf079, https://doi.org/10.1093/ckj/sfaf079

A correction has been made to the seventh-listed author's name.

