# Correction to: Cardiometabolic protein expression levels and pathways associated with kidney function decline in older European adults with advanced kidney disease

**DOI:** 10.1093/ckj/sfag310

**Published:** 2026-09-21

This is a correction to: Ryan E Aylward, Samantha Hayward, Nicholas C Chesnaye, Roemer J Janse, P Andreas Jonsson, Claudia Torino, Antonio Demetrio Vilasi, Maciej Szymczak, Christiane Drechsler, Friedo W Dekker, Marie Evans, Kitty J Jager, Christoph Wanner, Brian Rayner, Yoav Ben-Shlomo, Nicki Tiffin, Fergus J Caskey, Kate Birnie, for the EQUAL investigators, Cardiometabolic protein expression levels and pathways associated with kidney function decline in older European adults with advanced kidney disease, *Clinical Kidney Journal*, Volume 18, Issue 4, April 2025, sfaf079, https://doi.org/10.1093/ckj/sfaf079.

In the originally published version of this manuscript, the MRC grant code was given as ‘R103919-101’. This is incorrect and instead the code should read ‘MR/Y011139/1’.

These details have been corrected only in this correction notice to preserve the published version of record.

