# Supplementary material for: Cardiometabolic protein expression levels and pathways associated with kidney function decline in older European adults with advanced kidney disease
Source: Clin Kidney J. 2025 Mar 18;18(4):sfaf079. doi: 10.1093/ckj/sfaf079 (PMC12059643; doi:10.1093/ckj/sfaf079)
Supplement: sfaf079_Supplemental_File [file sfaf079_supplemental_file.docx]

**Cardiometabolic protein expression levels and pathways associated with kidney function decline in older European adults with advanced kidney disease**

*Supplementary methods and results*

Ryan E Aylward, Samantha Hayward, Nicholas C Chesnaye, Roemer J Janse, P. Andreas Jonsson, Claudia Torino, Antonio Demetrio Vilasi, Maciej Szymczak, Christiane Drechsler, Friedo W Dekker, Marie Evans, Kitty J Jager, Christoph Wanner, Brian Rayner, Yoav Ben-Shlomo, Nicki Tiffin, Fergus J Caskey and Kate Birnie, for the EQUAL investigators.

Contents

[1 Supplementary methods 2](#_Toc184288881)

[1.1 Olink® proteomic assay technology 4](#_Toc184288882)

[1.2 Sample handling and storage 4](#_Toc184288883)

[1.3 Motivation for the use of pathway enrichment analysis of regulatory pathways 4](#_Toc184288884)

[1.4 Joint model 4](#_Toc184288885)

[1.4.1 Longitudinal sub-model 5](#_Toc184288886)

[1.4.2 Time-to-event sub-model 5](#_Toc184288887)

[1.4.3 Software 6](#_Toc184288888)

[1.5 Missing data 6](#_Toc184288889)

[2 Supplementary Results 8](#_Toc184288890)

[2.1 Olink® protein assay characteristics 9](#_Toc184288891)

[2.2 Missing data and multiple imputation modelling 12](#_Toc184288892)

[2.3 Biological pathway principal component analysis 16](#_Toc184288893)

[3 References 22](#_Toc184288894)

[4 Protein abbreviations and names 24](#_Toc184288895)

# Supplementary methods


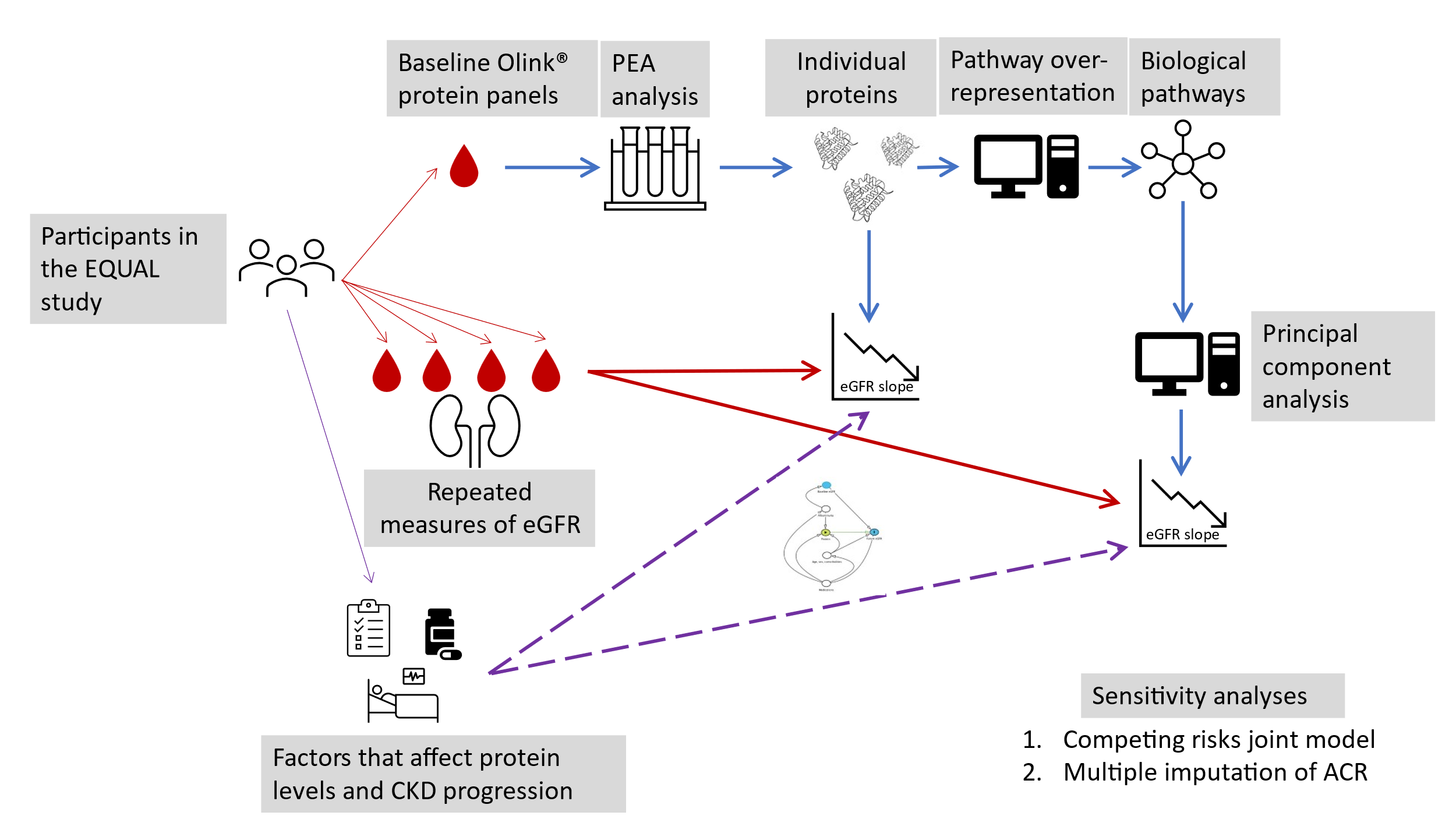


Fig S 1 Illustration of the analyses conducted in this study

Abbreviations: **PEA**, proximity extension assay; **EQUAL**, European Quality study; **eGFR**, estimated glomerular filtration rate; **CKD**, chronic kidney disease; **ACR**, albumin: creatinine ratio (urine).


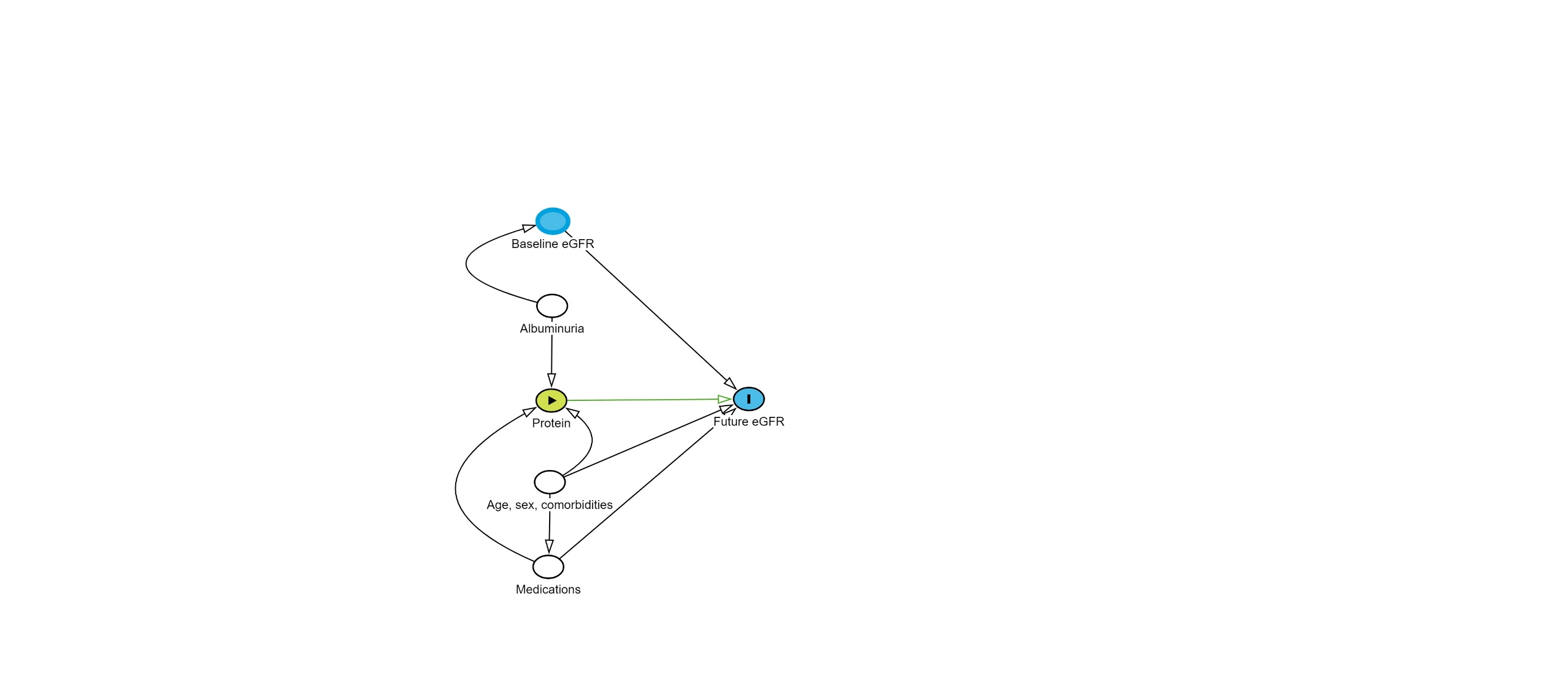


Fig S 2 Directed acyclic graph showing the postulated exposure-outcome relationship in the setting of other variables and biases

Nodes (variables) are connected by arrowed edges. The exposure(s) of interest were Olink® proteins and the outcome was eGFR slope. Blue nodes represent the only direct ancestors (causes) of the outcome, other than the exposure (green). The green edge represents the direct analysis of interest (front door). All back doors through the white nodes are blocked after adjustment. Age, sex, systolic blood pressure, diabetes status and primary renal disease are represented by the single ‘comorbidities’ node for simplicity, included from the baseline visit only (time-invariant). Please note the hidden arrowhead from ‘Medications’ to ‘Future eGFR’. Abbreviations: eGFR, estimated glomerular filtration rate. This DAG was produced using the DAGitty web-based environment.[1] Abbreviations: **eGFR**, estimate glomerular filtration rate.

## Olink® proteomic assay technology

In brief, the technology uses a Proximity Extension Assay to quantify protein concentrations that are reported on a log-2 scale as an arbitrary normalised protein eXpression (NPX) value. A one-unit increase can be interpreted as a doubling of protein concentration.

## Sample handling and storage

Plasma samples, collected in gel-separated tubes at the time of the baseline study visit, were refrigerated up to -80° Celsius at the local study site and subsequently shipped to a national laboratory in each country. Batches of samples were transferred to a biobank in Würzburg, Germany (for samples originating in UK, Poland, and Germany) or Karolinska Institutet biobank, Sweden (for samples originating in Sweden).

## Motivation for the use of pathway enrichment analysis of regulatory pathways

Gene Ontology annotations for the proteins of interest were explored to identify the particular biological pathways in which the proteins are active.[9] Since these Olink® protein panels were curated based on extant evidence of their involvement in cardiovascular disease, the proteins that a certain pathway represents may be associated with kidney function simply because the panel consisted of a sizeable number of similar proteins. For example, the analysed panels included eight complement cascade and 21 extracellular matrix organisation-related proteins.

## Joint model

The joint model framework includes two or more sub-models that are analysed simultaneously:

1. MLM longitudinal model (see main text) and,

2. Time-to-event model(s) which will be described below.

Parameters computed by the longitudinal model are incorporated into the estimation of the survival model and vice versa. The advantages of using JMs are to allow for the effects of potential informative censoring in the estimation of the longitudinal model by stipulating how the time to early dropout (by KRT initiation, death and LTFU) affects eGFR slope. Only the longitudinal model parameters i.e., the slope coefficient, were of interest and reported. A Poisson distribution family was used but robust standard errors were not available at the time of executing the current version of merlin.

### Longitudinal sub-model

A random intercept and slope were specified. Iterations of the longitudinal model sequentially adjusted included models 0 – 4.

### Time-to-event sub-model

The time from which eGFR measurements were recorded in the study i.e., the baseline visit, was taken to be when the study participant was first at risk in all time-to-event analyses and the timescale was measured in years until the first censoring event (either KRT initiation, death, LTFU or otherwise censored as alive at last follow-up).

Each competing risk component sub-model was included separately. In other words, in addition to the longitudinal MLM model, a sub-model for each time to KRT, death, LTFU and administrative censoring was specified. Administrative censoring occurred when end of follow-up was reached, or data extraction was undertaken and no occurrence of KRT initiation, death or LTFU had otherwise been experienced. The KRT time-to-event sub-model was adjusted for age, sex, country, and primary renal disease. The death time-to-event sub-model was adjusted for age, sex, diabetes mellitus status, systolic blood pressure, country, primary renal disease and ACR.

### Software

MJ Crowther’s merlin v2.1.5 user-written command was used to execute the joint model (Mixed Effects Regression for LInear, Non-linear and user-defined models).[2] Robust standard errors were not available in this version.

## Missing data

There were some missing data for Olink® proteins. Superoxide dismutase 1 protein failed quality control in 97 individuals in the discovery sub-cohort. None were missing protein data in the validation sub-cohort. Age, sex and country had zero missing values. Information was missing for <1% for the co-variables diabetes mellitus status, primary renal disease and systolic blood pressure. Estimated GFR (the outcome) was missing for <1% and missingness of the repeated measure variable is inherently handled within the multilevel model. Of most concern, baseline ACR was missing in 43%. Five per cent of missing values were recovered by converting urinary protein: creatinine ratio to ACR using Weaver’s equation.[3] Data were collected from patient questionnaires at the time of routine clinic visits.[4] Biochemical results were recorded from medical records at these associated visits and testing was not specifically requested for the purpose of the EQUAL study. It would be expected that every non-anuric patient visiting the kidney clinic would undergo a urine test (at the very least a urine dipstick) and no one should be expected to be anuric at the baseline visit with an eGFR of 20 mL/min/1.73m^2^ and so be unable to produce urine. Proteinuria would therefore be readily detected and a urine ACR or protein:creatinine ratio requested, and its result recorded. It was hypothesised that significant proteinuria would be more likely to be detected and recorded and so its missingness was dependent on unobserved proteinuria levels. Alternatively, urine collection or result recording may have differed because of site-specific practice patterns.


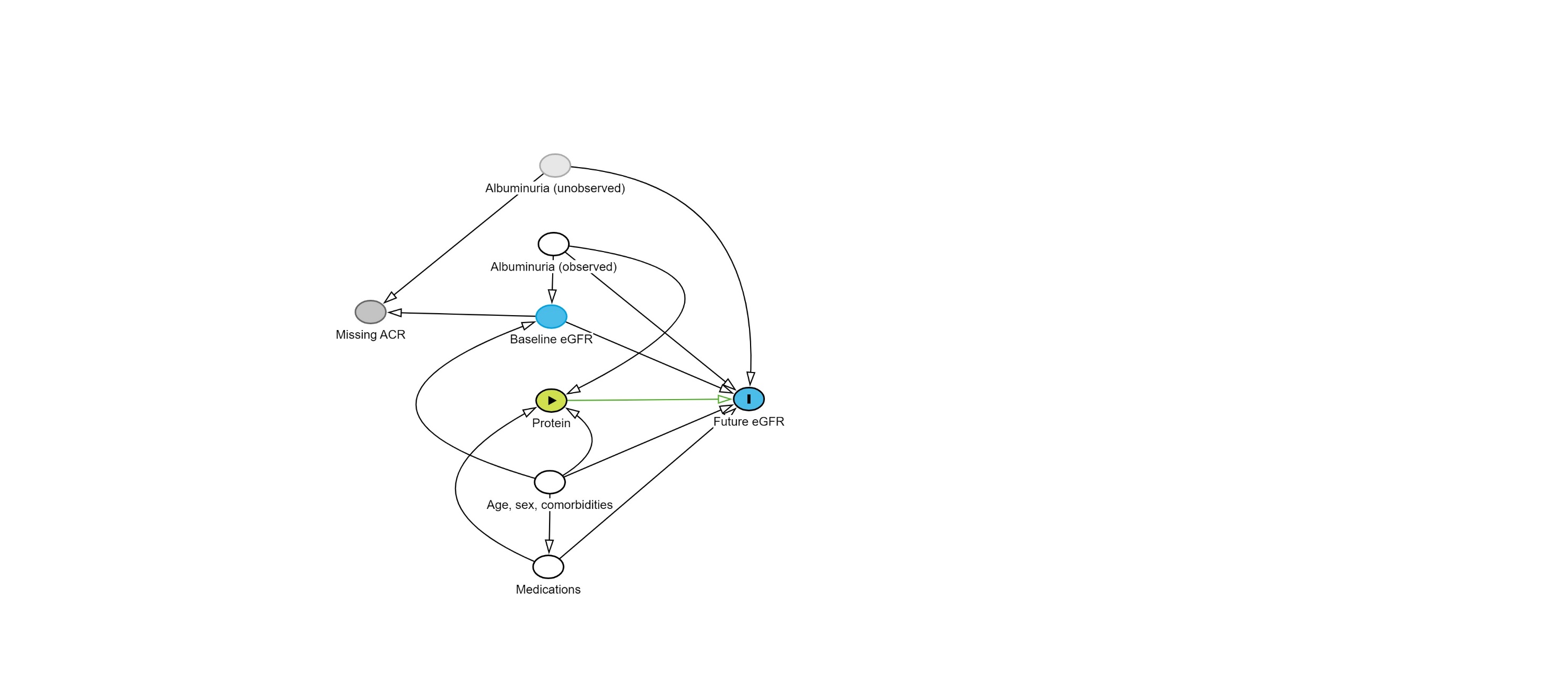


Fig S 3 Potential missing data mechanism for albuminuria visualised as a directed acyclic graph

In this DAG, the assumed relationship between the exposure (green), adjusted for independent variables (white), outcome (blue) and unobserved variables (grey) are shown. There is no relationship between the outcome and the chance of having a missing ACR. This assumption was corroborated by a logistic regression analysis showing that the odds of being a complete case was no higher for varying levels of eGFR slope. Note the hidden arrow from ‘Medications’ to ‘Future **eGFR’**. Abbreviations: **ACR**, albumin to creatinine ratio; **eGFR**, estimated glomerular filtration rate. Created using DAGitty.[1]

# Supplementary Results


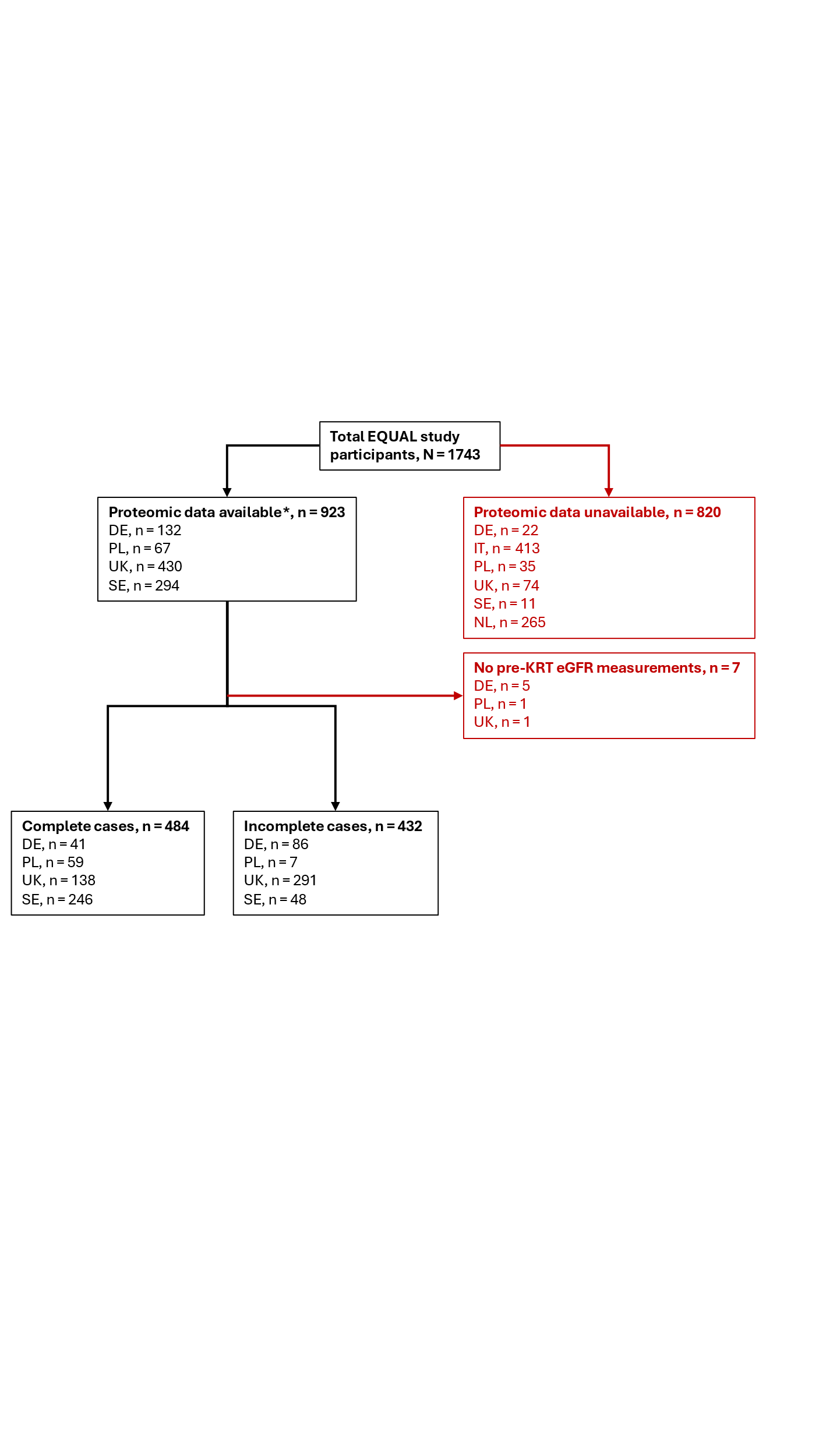


Fig S 4 Flow diagram of included and excluded participants

A total of 1743 participants were enrolled in the EQUAL study. * Proteomic data were only available for participants from Germany, Poland, The United Kingdom and Sweden. Abbreviations: **DE**, Germany; **IT**, Italy; **PL**, Poland; **UK**, The United Kingdom; **SE**, Sweden; **NL**, The Netherlands; **KRT**, kidney replacement therapy; **eGFR**, estimated glomerular filtration rate.


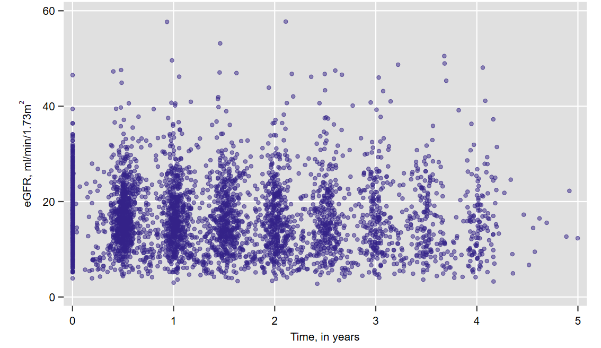


Fig S 5 Scatterplot of eGFR over follow-up time

eGFR was calculated using the CKD-EPI 2009 equation. Abbreviations: **eGFR**, estimated glomerular filtration rate; **CKD-EPI**, Chronic Kidney Disease Epidemiology.

## Olink® protein assay characteristics

The intra- and inter-assay Coefficient of Variance (CV) for the two protein panels (Target 96 cardiometabolic and Target 96 cardiovascular II) for the discovery sub-cohort was 6 – 7% and 12 – 19% respectively, and 5 – 6% (intra-assay) and 15% (inter-assay) for the validation sub-cohort. An intra-assay CV of < 15% and an inter-assay CV of < 25% are considered acceptable by Olink®. Laboratory validation of the protein panels can be found at <https://olink.com/resources-support/document-download-center/>.


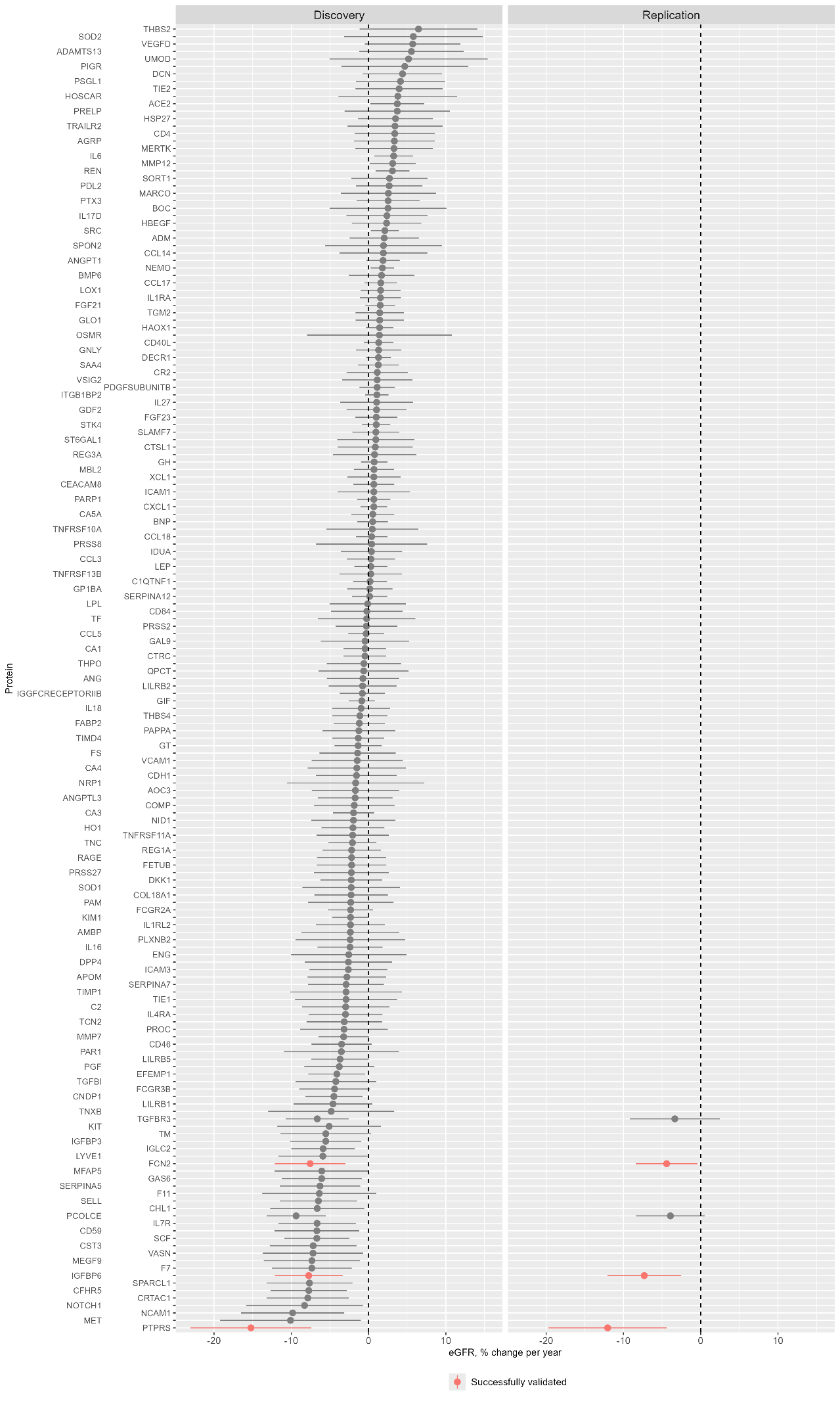


Fig S 6 Forest plot of the protein-slope associations estimated in discovery and validation sub-cohorts

A protein abbreviation list is available at the end of the supplementary material.

## Missing data and multiple imputation modelling

|  | **Complete cases**, n = 484 | **Incomplete cases**, n = 432 |
| --- | --- | --- |
| **Age in years**, median (IQR) | 75 (70, 80) | 77 (72, 82) |
| **Female sex**, % | 33% | 39% |
| **Country**, %  Germany  United Kingdom  Poland  Sweden | 8.5%  29%  12%  51% | 20%  67%  1.6%  11% |
| **Comorbidities**, Yes %  Diabetes mellitus  Hypertension  Coronary artery disease  Heart failure | 40%  88%  36%  19% | 43%  83%  23%  14% |
| **Primary renal disease**, %  Glomerular  Tubulo-interstitial  Diabetes  Renovascular  Other systemic disease  Hereditary  Miscellaneous | 11%  9.9%  23%  35%  3.7%  4.3%  13% | 9.2%  9.2%  20%  31%  1.9%  3.8%  25% |
| **Clinical measurements**  Current smoking, Yes %  Systolic blood pressure mmHg, median (IQR)  BMI kg/m^2^, median (IQR) | 7.1%  146 (131, 160)  27.6 (24.5, 31.2) | 8.3%  144 (130, 160)  28.7 (25.4, 32.9) |
| **N of eGFR values per person**, median (IQR)  Total N of eGFR values | 7 (5, 9)  2,494 | 5 (4, 8)  1,978 |
| **Laboratory measurements**, median (IQR)  eGFR CKD-EPI, mL/min/1.73m^2^  Calcium, mmol/L  Phosphate, mmol/L  Parathyroid hormone, pmol/L  Total cholesterol, mmol/L | 17.6 (14.7, 20.8)  2.27 (2.17, 2.37)  1.29 (1.12, 1.46)  16 (10, 23)  4.60 (3.80, 5.50) | 17.4 (13.7, 21)  2.29 (2.18, 2.38)  1.27 (1.10, 1.45)  16 (8, 25)  4.27 (3.40, 5.27) |

Table S 1 Comparison of baseline characteristics of participants with and without complete data at baseline

Abbreviations: **BMI**, body mass index; **eGFR**, estimated glomerular filtration rate; **ACR**, albumin: creatinine ratio; **IQR**, interquartile range.

Patients with complete and incomplete data are compared in Table S 1; most notable was the unequal study country distribution. Even after adjustment for primary renal disease (stricter monitoring of ACR may have been expected in individuals with glomerulonephritides or diabetic nephropathy diagnoses) or country (as a proxy of local testing practices), and other variables, the outcome (slope) did not increase the odds of ACR being unobserved (OR 0.71; 95% CI 0.42, 1.18; P = 0.187).

The multiple imputation model identified additional proteins that may be associated with eGFR decline and computed similar slope estimates as the primary analysis for PTPRS, IGFBP6 and FCN2. The additional proteins, barring CST3 an F7, demonstrate very similar extracellular and immunological functions.

Most relevant to those already discussed, EFEMP1 was associated with eGFR decline in a Mendelian randomisation study in participants of the Framingham Heart Offspring cohort which adds to the finding of fibrosis pathways as a cause of CKD progression.[5] Immunoglobulin light chains, which IGLC2 is a component of, is profibrotic by activating reactive oxygen system pathways in in vivo experiments.[6] NCAM1, a neuronal cell adhesion molecule, may act as an autoantigen in membranous lupus nephritis and has been found expressed in fibrosed human interstitum.[7,8] Also, serum levels of PCOLCE, was found to be higher in people with CKD compared to those with normal kidney function.[9] Further, CD59, a protein that regulates the membrane attack complex which is the final product of the complement cascade, was less expressed on human leukocytes in people with CKD to possibly counter complement hyperactivity.[10]

Also, CKD is a pro-coagulable state and several haemostatic components, including F7 that was identified in this analysis, have been found to increase as CKD progresses.[11,12] Finally, CST3 (cystatin-C) is a well-known biomarker of kidney function and improves the prediction of progression to KF, cardiovascular events and death compared to creatinine based eGFR equations, especially in older people in whom CST3 is less affected by muscle mass.[13,14] Unfortunately CST3 was not assayed longitudinally so could not be incorporated into the eGFR calculation.


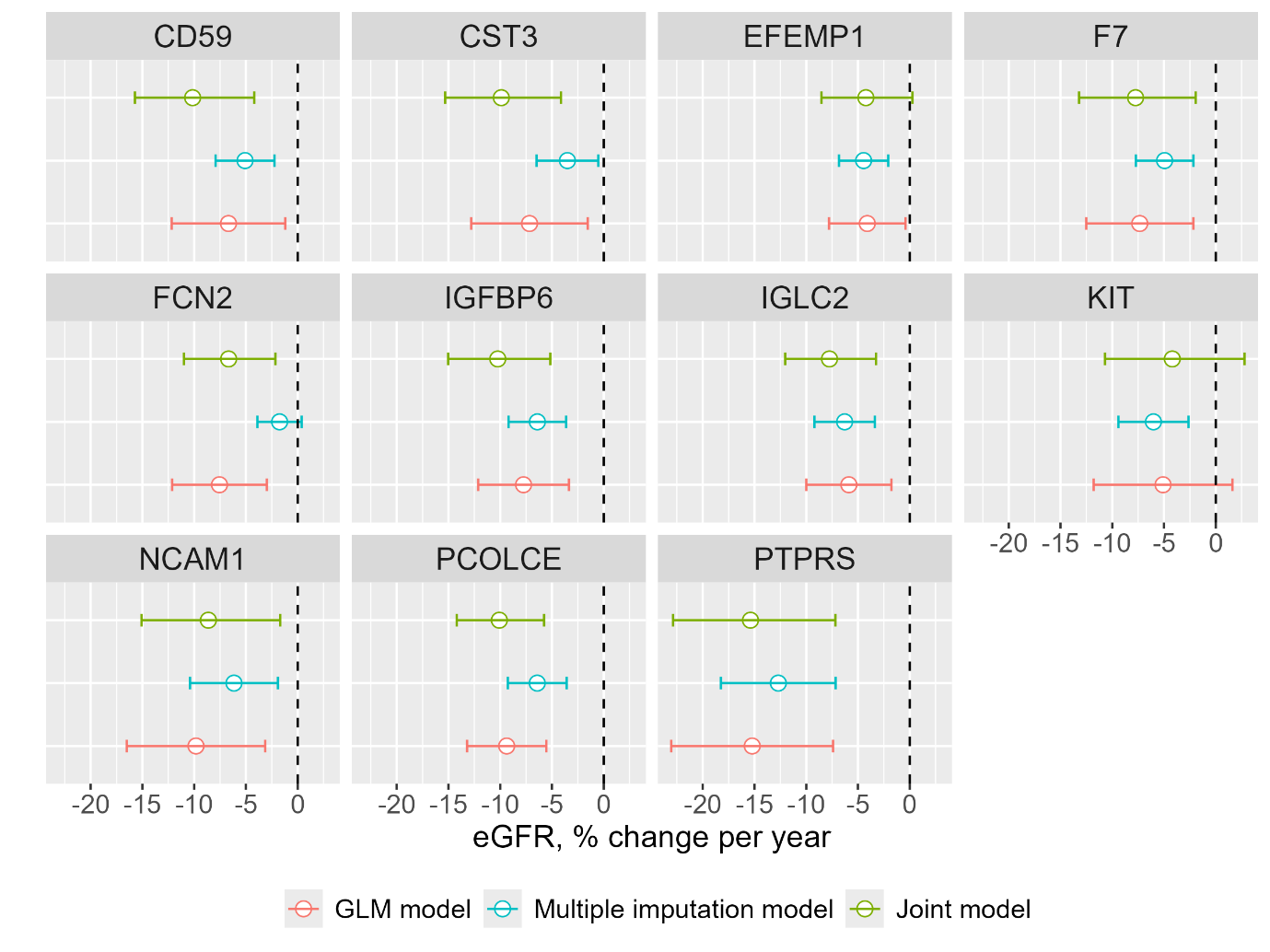


Fig S 7 Slope estimates compared for the primary, imputation and joint models

The slope estimates shown are for all proteins successfully validated in each of the primary GLM, multiple imputation and joint models. Only estimates for the discovery sub-cohort are illustrated here as the same proteins were not all taken to validation in the respective validation analyses. The computed slope estimates differ for each model type, but the 95% confidence intervals overlap. This suggests that there was negligible effect of informative censoring, but the multiple imputation model did offer improvements in estimate precision. Abbreviations: **GLM**, generalised linear mixed effects; **eGFR**, estimated glomerular filtration rate; **CD59**, cluster of differentiation 59; **CST3**, cystatin 3/ cystatin C; **EFEMP1**, EGF-containing fibulin-like extracellular matrix protein 1; **F7**, clotting factor VII; **FCN2**, ficolin 2; **IGFBP6**, insulin-like growth factor binding protein 6; **IGLC2**, Immunoglobulin light chain constant 2; **KIT**, mast/ stem cell factor receptor kit; **NCAM1**, neural cell adhesion molecule 1; **PCOLCE**, procollagen C-endopeptidase enhancer 1; **PTPRS**, receptor-type tyrosine-protein phosphatase S.

## Biological pathway principal component analysis


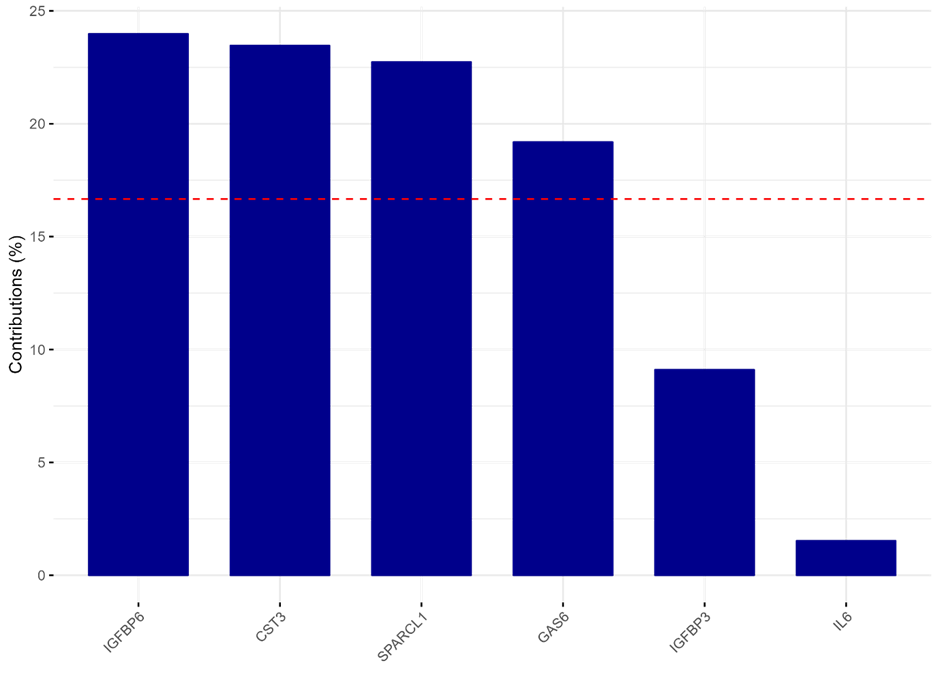

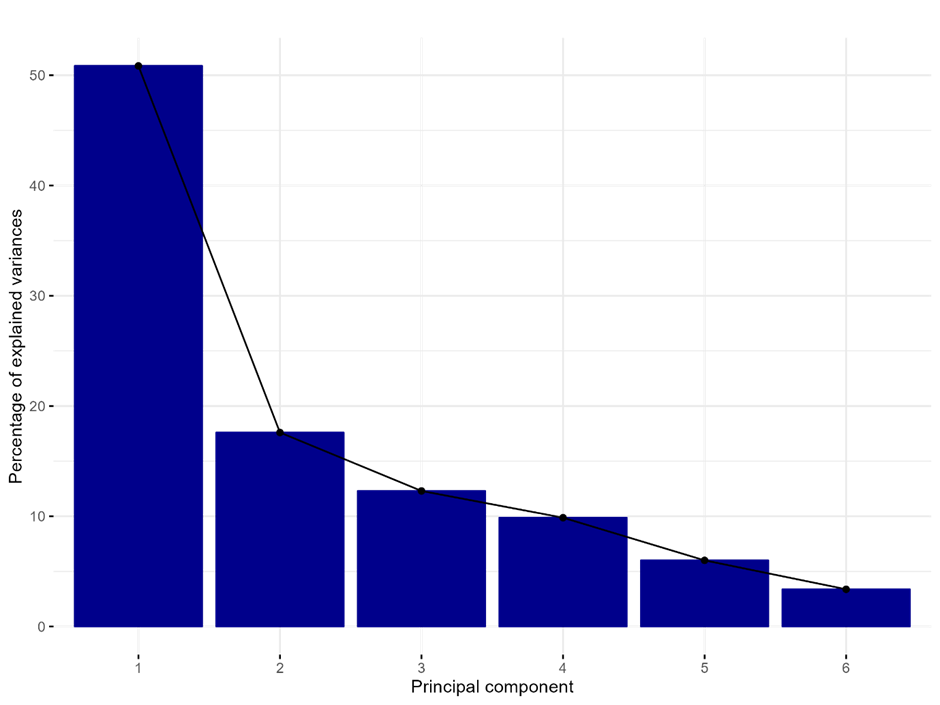


**B**

**A**

Fig S 8 Regulation of IGF transport and uptake by binding proteins biological pathway principal component analysis

(A) shows the contribution of each protein to the first principal component. The dashed red line is the mean contribution. (B) illustrates the scree plot that is visually inspected to determine the number of principal components that maximally summarise the variation in the biological pathway as a group. Abbreviations: **IGFBP6**, Insulin-like growth factor-binding protein 6; **CST3**, Cystatin-C; **SPARCl1**, SPARC-like protein 1; **GAS6**, Growth arrest-specific protein 6; **IGFBP3**, Insulin-like growth factor-binding protein 3; **IL6**, Interleukin 6.


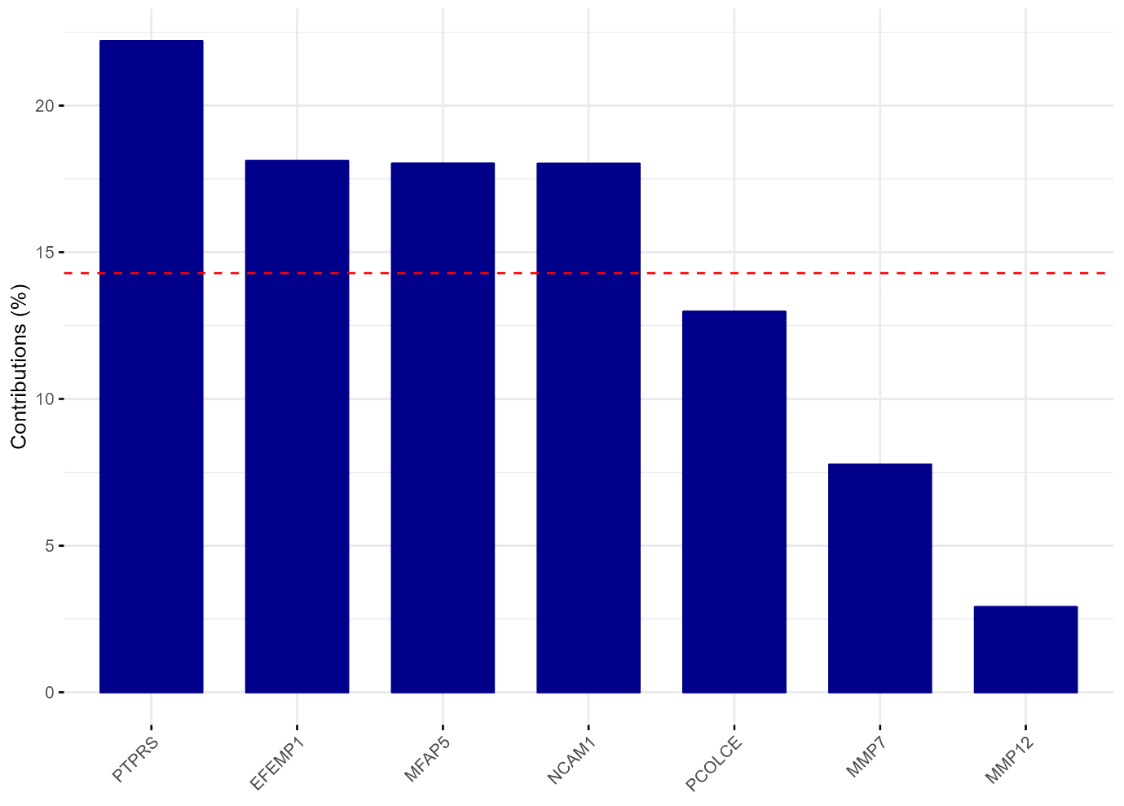


**A**


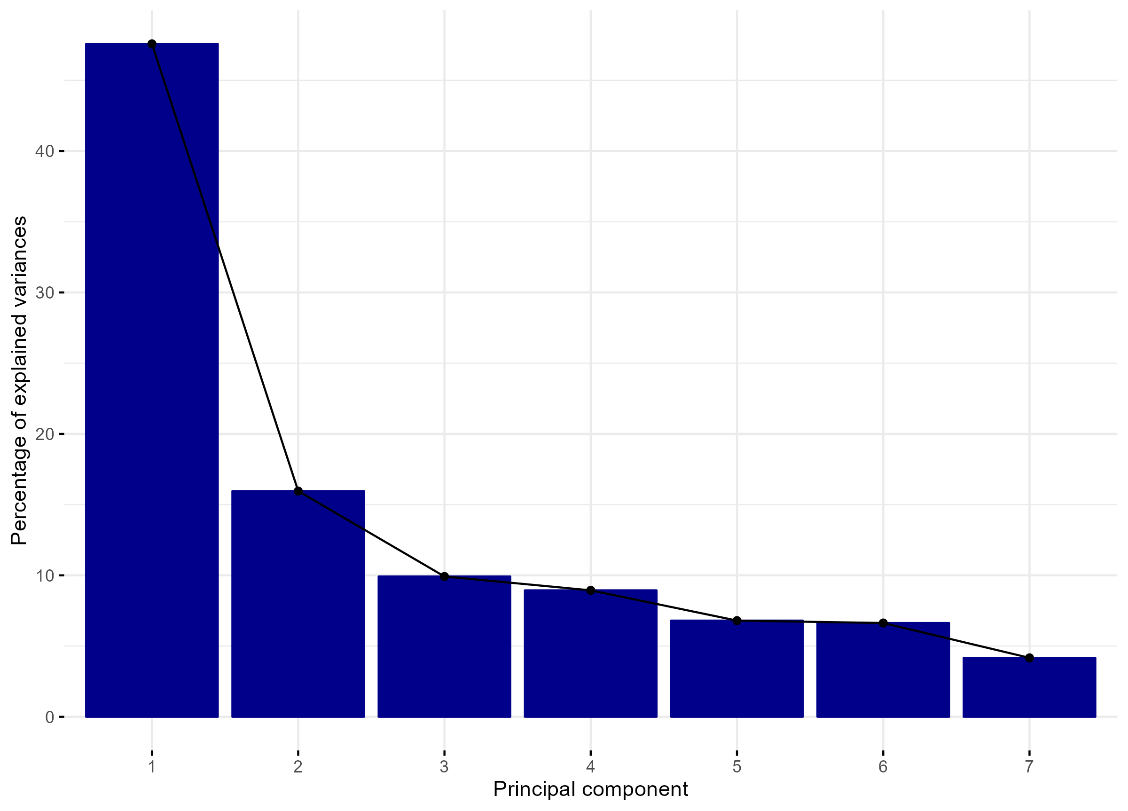


**B**

Fig S 9 Extracellular matrix organisation biological pathway principal component analysis

(A) shows the contribution of each protein to the first principal component. The dashed red line is the mean contribution. (B) illustrates the scree plot that is visually inspected to determine the number of principal components that maximally summarise the variation in the biological pathway as a group. Abbreviations: **PTPRS**, Receptor-type tyrosine-protein phosphatase S; **EFEMP1**, EGF-containing fibulin-like extracellular matrix protein 1; **MFAP5**, Microfibrillar-associated protein 5; **NCAM1**, Neural cell adhesion molecule 1; **PCOLCE**, Procollagen C-endopeptidase enhancer 1; **MMP7**, Matrix metalloproteinase-7; **MMP12**, Matrix metalloproteinase-12.


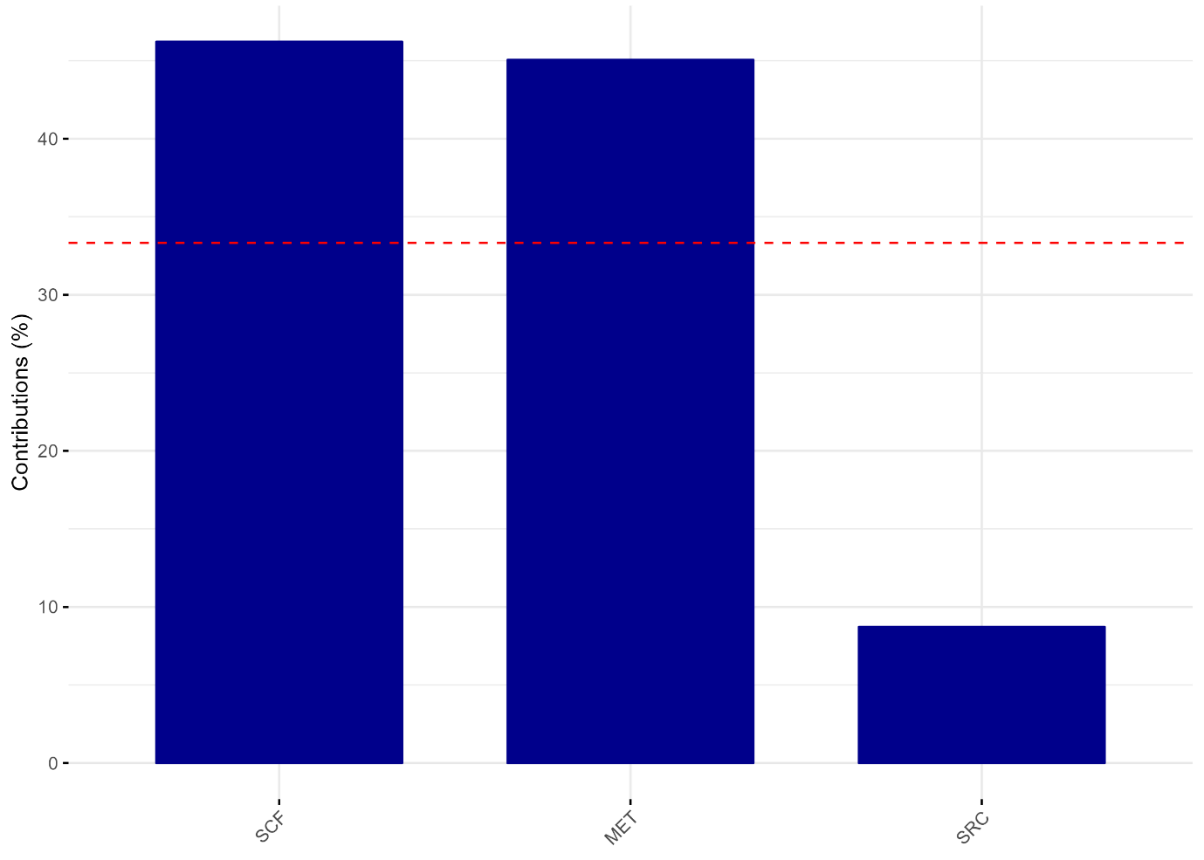

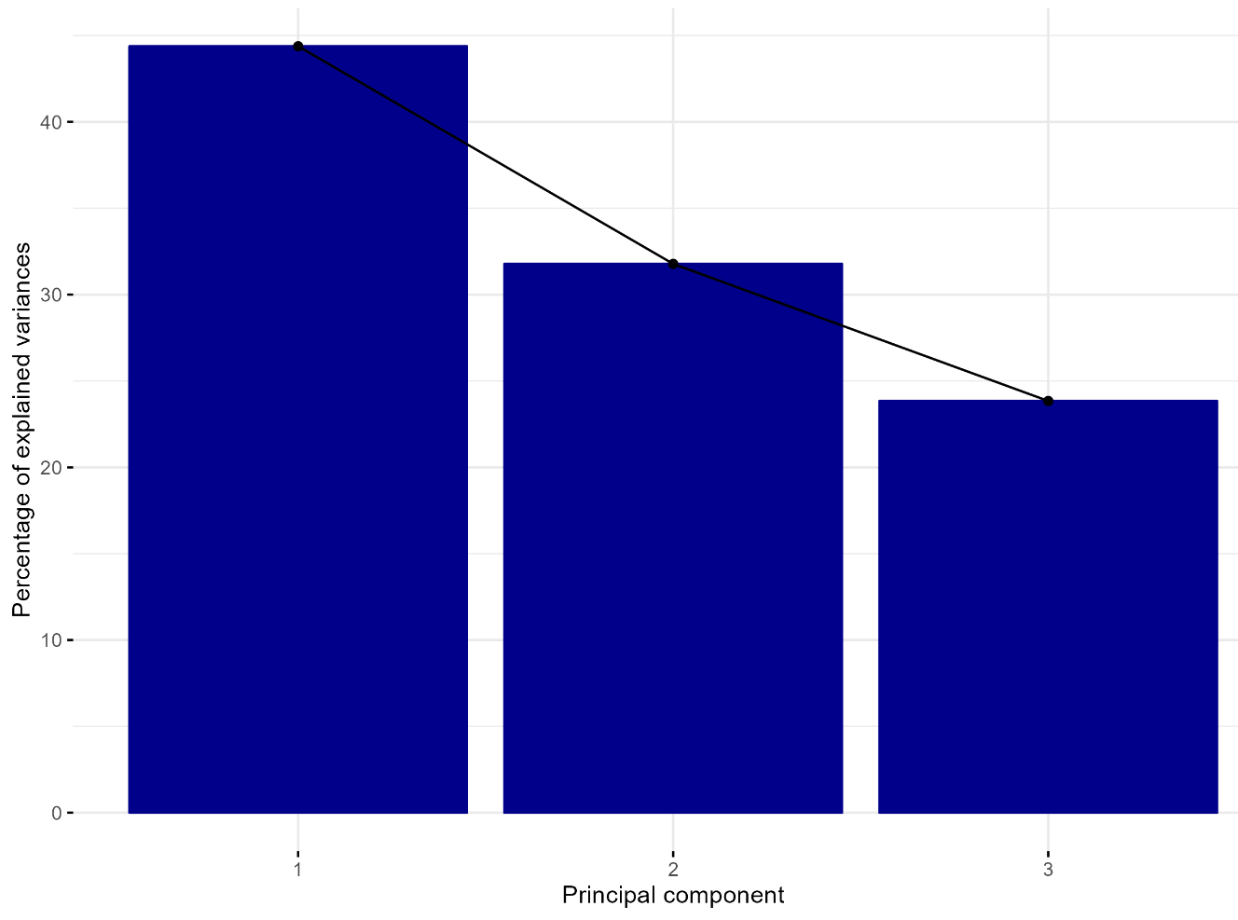


**B**

**A**

Fig S 10 PI3K/ Akt signalling biological pathway principal component analysis

(A) shows the contribution of each protein to the first principal component. The dashed red line is the mean contribution. (B) illustrates the scree plot that is visually inspected to determine the number of principal components that maximally summarise the variation in the biological pathway as a group. Abbreviations: **SCF**, Kit ligand/ stem cell factor; **MET**, hepatocyte growth factor receptor; **SRC**, proto-oncogene tyrosine-protein kinase Src.


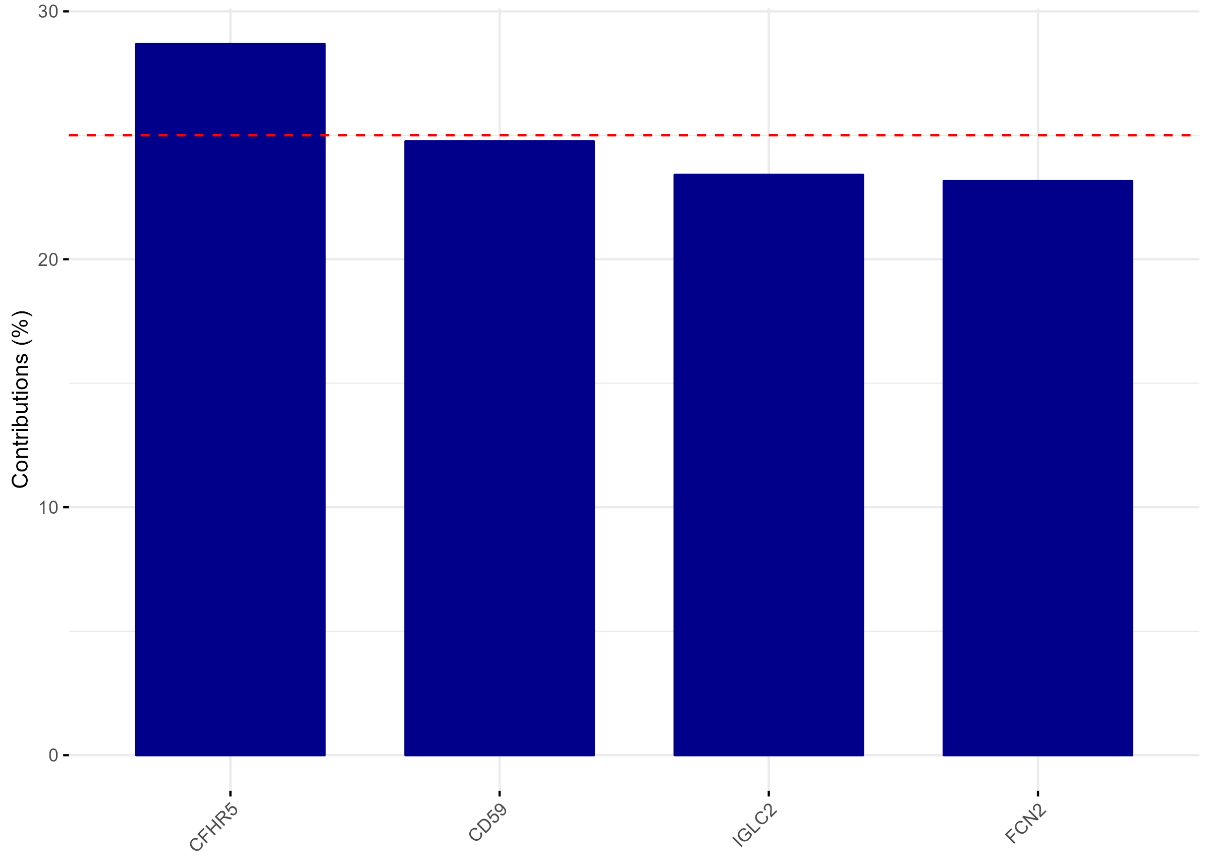

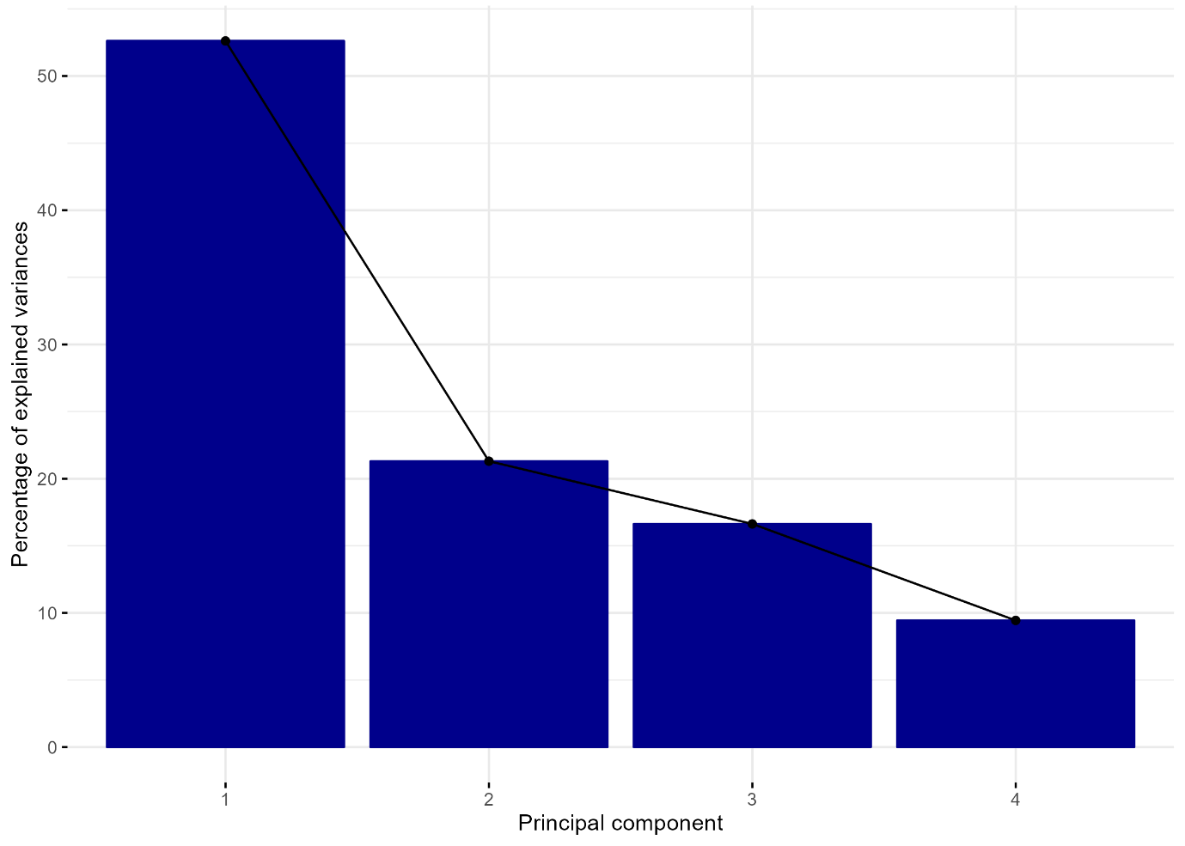


**B**

**A**

Fig S 11 Complement cascade biological pathway principal component analysis

(A) shows the contribution of each protein to the first principal component. The dashed red line is the mean contribution. (B) illustrates the scree plot that is visually inspected to determine the number of principal components that maximally summarise the variation in the biological pathway as a group. Abbreviations: **CFHR5**, complement factor H-related protein 5; **CD59**, cluster of differentiation 59; **IGLC2**, Immunoglobulin lambda constant 2; **FCN2**, ficolin 2.


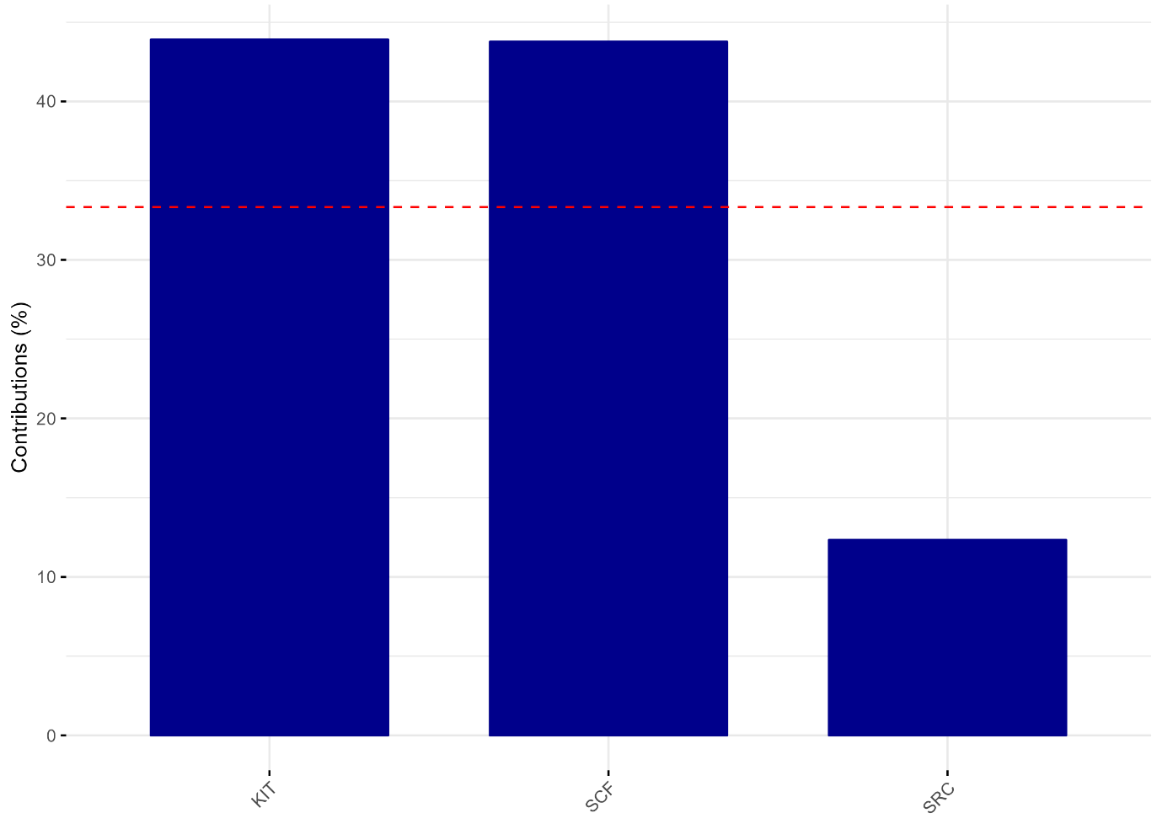

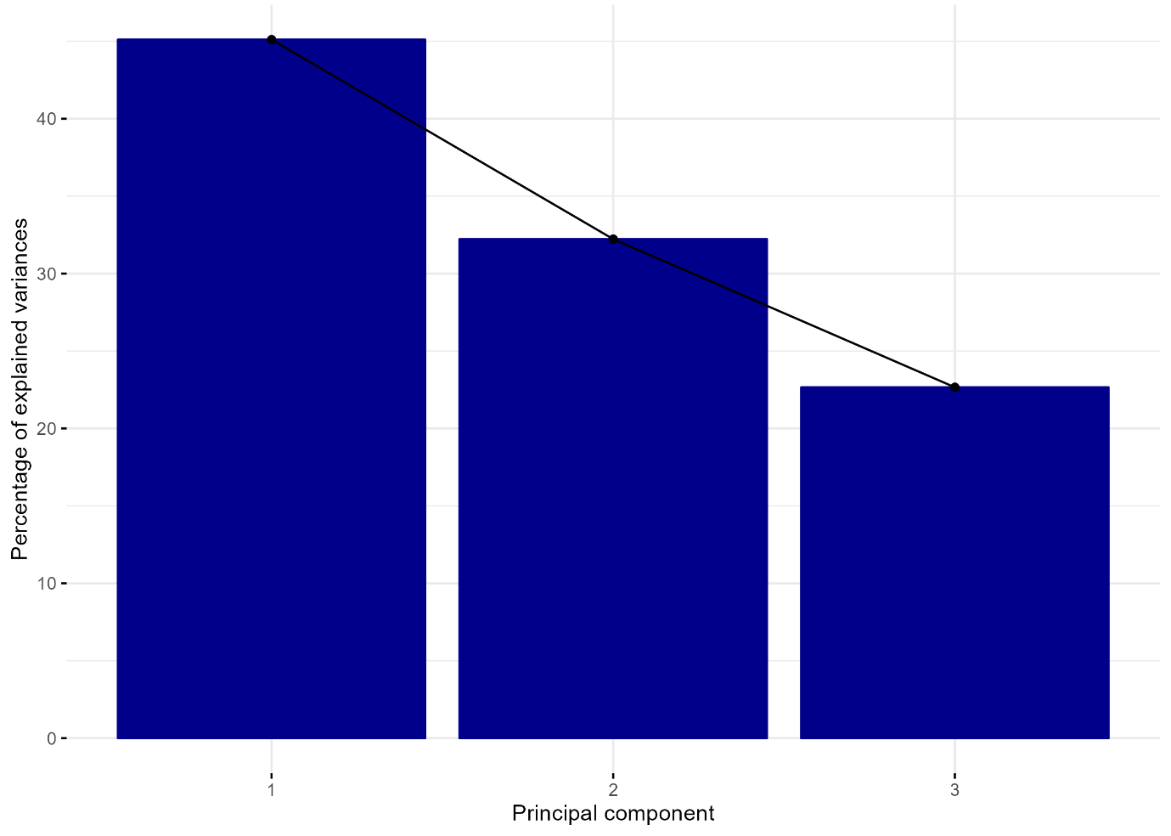


**B**

**A**

Fig S 12 KIT signalling biological pathway principal component analysis

(A) shows the contribution of each protein to the first principal component. The dashed red line is the mean contribution. (B) illustrates the scree plot that is visually inspected to determine the number of principal components that maximally summarise the variation in the biological pathway as a group. Abbreviations: **KIT**, mast/stem cell factor receptor kit; **SCF**, Kit ligand/ stem cell factor; **SRC**, proto-oncogene tyrosine-protein kinase Src.


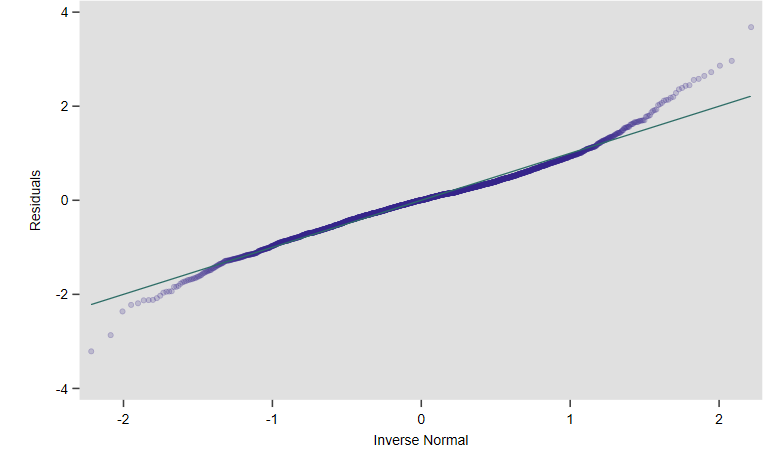


Fig S 13 Quantile-Quantile plot showing the distribution of model residuals

The residuals were approximately normally distributed as they generally follow the line of equality indicating an overall good model fit, except at the extremes where some curve was noted.


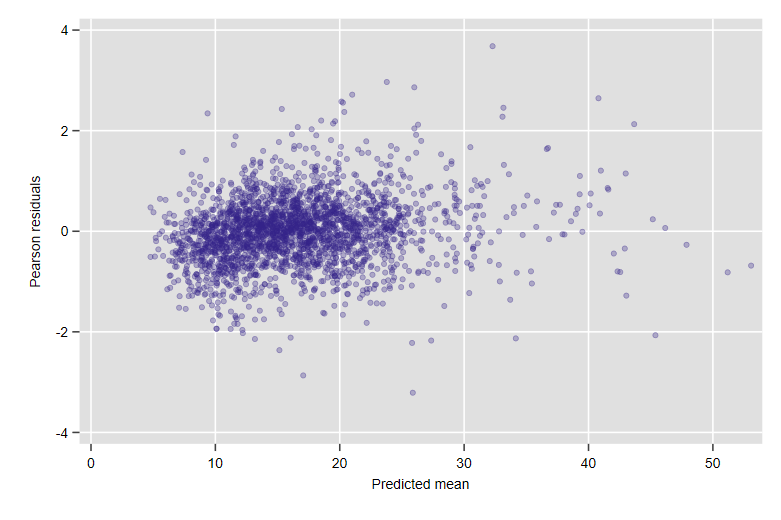


Fig S 14 Model residuals versus fitted values as a check for heteroscedasticity

Towards the left of the plot, points are grouped in no particular pattern. The data points on the right are less dense but still do not show an obvious relationship between the model residuals and fitted values indicating a low likelihood of heteroscedasticity.

# References

1. Textor J, Hardt J, Knüppel S. DAGitty: A Graphical Tool for Analyzing Causal Diagrams. Epidemiology. 2011 Sep;22(5):745.

2. Crowther MJ. merlin—A unified modeling framework for data analysis and methods development in Stata. Stata J. 2020 Dec 1;20(4):763–84.

3. Weaver RG, James MT, Ravani P, Weaver CGW, Lamb EJ, Tonelli M, et al. Estimating Urine Albumin-to-Creatinine Ratio from Protein-to-Creatinine Ratio: Development of Equations using Same-Day Measurements. J Am Soc Nephrol JASN. 2020 Mar;31(3):591–601.

4. Jager KJ, Ocak G, Drechsler C, Caskey FJ, Evans M, Postorino M, et al. The EQUAL study: A European study in chronic kidney disease stage 4 patients. Nephrol Dial Transplant. 2012;27(SUPPL. 3):27–31.

5. Keshawarz A, Hwang SJ, Lee GY, Yu Z, Yao C, Köttgen A, et al. Cardiovascular disease protein biomarkers are associated with kidney function: The Framingham Heart Study. PloS One. 2022;17(5):e0268293.

6. Ying WZ, Li X, Rangarajan S, Feng W, Curtis LM, Sanders PW. Immunoglobulin light chains generate proinflammatory and profibrotic kidney injury. J Clin Invest. 129(7):2792–806.

7. Caza TN, Hassen SI, Kuperman M, Sharma SG, Dvanajscak Z, Arthur J, et al. Neural cell adhesion molecule 1 is a novel autoantigen in membranous lupus nephritis. Kidney Int. 2021 Jul 1;100(1):171–81.

8. Marković-Lipkovski J, Životić M, Müller CA, Tampe B, Ćirović S, Vještica J, et al. Variable Expression of Neural Cell Adhesion Molecule Isoforms in Renal Tissue: Possible Role in Incipient Renal Fibrosis. PloS One. 2015;10(9):e0137028.

9. Özkan G, Güzel S, Atar RV, Fidan Ç, Kara SP, Ulusoy Ş. Elevated serum levels of procollagen C-proteinase enhancer-1 in patients with chronic kidney disease is associated with a declining glomerular filtration rate. Nephrol Carlton Vic. 2019 Sep;24(9):938–42.

10. Eldewi DM, Alhabibi AM, El Sayed HME, Mahmoud SAK, El Sadek SM, Gouda RM, et al. Expression levels of complement regulatory proteins (CD35, CD55 and CD59) on peripheral blood cells of patients with chronic kidney disease. Int J Gen Med. 2019 Sep 16;12:343–51.

11. Dubin R, Cushman M, Folsom AR, Fried LF, Palmas W, Peralta CA, et al. Kidney function and multiple hemostatic markers: cross sectional associations in the multi-ethnic study of atherosclerosis. BMC Nephrol. 2011 Jan 26;12(1):3.

12. Huang MJ, Wei R bao, Wang Y, Su T yu, Di P, Li Q ping, et al. Blood coagulation system in patients with chronic kidney disease: a prospective observational study. BMJ Open. 2017 Jun 1;7(5):e014294.

13. Peralta CA, Shlipak MG, Judd S, Cushman M, McClellan W, Zakai NA, et al. Detection of Chronic Kidney Disease With Creatinine, Cystatin C, and Urine Albumin-to-Creatinine Ratio and Association With Progression to End-Stage Renal Disease and Mortality. JAMA. 2011 Apr 20;305(15):1545–52.

14. Shlipak MG, Matsushita K, Ärnlöv J, Inker LA, Katz R, Polkinghorne KR, et al. Cystatin C versus creatinine in determining risk based on kidney function. N Engl J Med. 2013 Sep 5;369(10):932–43.

# Protein abbreviations and names

*Proteins that were not analysed due to missingness.

| **Short name** | **UniProt ID** | **Long name** | **Olink® panel name** |
| --- | --- | --- | --- |
| ACE2 | Q9BYF1 | Angiotensin-converting enzyme 2 | CVII |
| ADAM-TS13 | Q76LX8 | A disintegrin and metalloproteinase with thrombospondin motifs 13 | CVII |
| ADM | P35318 | ADM | CVII |
| AGRP | O00253 | Agouti-related protein | CVII |
| AMBP | P02760 | Protein AMBP | CVII |
| ANG | P03950 | Angiogenin | CM |
| ANG-1 | Q15389 | Angiopoietin-1 | CVII |
| ANGPTL3 | Q9Y5C1 | Angiopoietin-related protein 3 | CM |
| AOC3 | Q16853 | Membrane primary amine oxidase | CM |
| APOM | O95445 | Apolipoprotein M | CM |
| BMP-6 | P22004 | Bone morphogenetic protein 6 | CVII |
| BNP | P16860 | Natriuretic peptides B | CVII |
| C1QTNF1 | Q9BXJ1 | Complement C1q tumour necrosis factor-related protein 1 | CM |
| C2 | P06681 | Complement C2 | CM |
| CA1 | P00915 | Carbonic anhydrase 1 | CM |
| CA3 | P07451 | Carbonic anhydrase 3 | CM |
| CA4 | P22748 | Carbonic anhydrase 4 | CM |
| CA5A | P35218 | Carbonic anhydrase 5A, mitochondrial | CVII |
| CCL14 | Q16627 | C-C motif chemokine 14 | CM |
| CCL17 | Q92583 | C-C motif chemokine 17 | CVII |
| CCL18 | P55774 | C-C motif chemokine 18 | CM |
| CCL3 | P10147 | C-C motif chemokine 3 | CVII |
| CCL5 | P13501 | C-C motif chemokine 5 | CM |
| CD4 | P01730 | T-cell surface glycoprotein CD4 | CVII |
| CD40-L | P29965 | CD40 ligand | CVII |
| CD46 | P15529 | Membrane cofactor protein | CM |
| CD59 | P13987 | CD59 glycoprotein | CM |
| CD84 | Q9UIB8 | SLAM family member 5 | CVII |
| CDH1 | P12830 | Cadherin-1 | CM |
| CEACAM8 | P31997 | Carcinoembryonic antigen related cell adhesion molecule 8 | CVII |
| CES1* | P23141 | Liver carboxylesterase 1 | CM |
| CFHR5 | Q9BXR6 | Complement factor H-related protein 5 | CM |
| CHL1 | O00533 | Neural cell adhesion molecule L1-like protein | CM |
| CNDP1 | Q96KN2 | Beta-Ala-His dipeptidase | CM |
| COL18A1 | P39060 | Collagen alpha-1(XVIII) chain | CM |
| COMP | P49747 | Cartilage oligomeric matrix protein | CM |
| CR2 | P20023 | Complement receptor type 2 | CM |
| CRTAC1 | Q9NQ79 | Cartilage acidic protein 1 | CM |
| CST3 | P01034 | Cystatin-C | CM |
| CTRC | Q99895 | Chymotrypsin C | CVII |
| CTSL1 | P07711 | Cathepsin L1 | CVII |
| CXCL1 | P09341 | C-X-C motif chemokine 1 | CVII |
| DCN | P07585 | Decorin | CVII |
| DECR1 | Q16698 | 2,4-dienoyl-CoA reductase, mitochondrial | CVII |
| DEFA1* | P59665 | Neutrophil defensin 1 | CM |
| Dkk-1 | O94907 | Dickkopf-related protein 1 | CVII |
| DPP4 | P27487 | Dipeptidyl peptidase 4 | CM |
| EFEMP1 | Q12805 | EGF-containing fibulin-like extracellular matrix protein 1 | CM |
| ENG | P17813 | Endoglin | CM |
| F11 | P03951 | Coagulation factor XI | CM |
| F7 | P08709 | Coagulation factor VII | CM |
| FABP2 | P12104 | Fatty acid-binding protein, intestinal | CVII |
| FAP* | Q12884 | Prolyl endopeptidase FAP | CM |
| FCGR2A | P12318 | Low affinity immunoglobulin gamma Fc region receptor II-a | CM |
| FCGR3B | O75015 | Low affinity immunoglobulin gamma Fc region receptor III-B | CM |
| FCN2 | Q15485 | Ficolin-2 | CM |
| FETUB | Q9UGM5 | Fetuin-B | CM |
| FGF-21 | Q9NSA1 | Fibroblast growth factor 21 | CVII |
| FGF-23 | Q9GZV9 | Fibroblast growth factor 23 | CVII |
| FS | P19883 | Follistatin | CVII |
| Gal-9 | O00182 | Galectin-9 | CVII |
| GAS6 | Q14393 | Growth arrest-specific protein 6 | CM |
| GDF-2 | Q9UK05 | Growth/differentiation factor 2 | CVII |
| GH | P01241 | Growth hormone | CVII |
| GIF | P27352 | Gastric intrinsic factor | CVII |
| GLO1 | Q04760 | Lactoyl glutathione lyase | CVII |
| GNLY | P22749 | Granulysin | CM |
| GP1BA | P07359 | Platelet glycoprotein Ib alpha chain | CM |
| GT | P51161 | Gastrotropin | CVII |
| HAOX1 | Q9UJM8 | Hydroxyacid oxidase 1 | CVII |
| HB-EGF | Q99075 | Proheparin-binding EGF-like growth factor | CVII |
| HO-1 | P09601 | Heme oxygenase 1 | CVII |
| hOSCAR | Q8IYS5 | Osteoclast-associated immunoglobulin-like receptor | CVII |
| HSP 27 | P04792 | Heat shock 27 kDa protein | CVII |
| ICAM1 | P05362 | Intercellular adhesion molecule 1 | CM |
| ICAM3 | P32942 | Intercellular adhesion molecule 3 | CM |
| IDUA | P35475 | Alpha-L-iduronidase | CVII |
| IGFBP3 | P17936 | Insulin-like growth factor-binding protein 3 | CM |
| IGFBP6 | P24592 | Insulin-like growth factor-binding protein 6 | CM |
| IGLC2 | P0DOY2 | Ig lambda-2 chain C regions | CM |
| IL-17D | Q8TAD2 | Interleukin-17D | CVII |
| IL-18 | Q14116 | Interleukin-18 | CVII |
| IL-1ra | P18510 | Interleukin-1 receptor antagonist protein | CVII |
| IL-27 | Q14213 | Interleukin-27 | CVII |
| IL-4RA | P24394 | Interleukin-4 receptor subunit alpha | CVII |
| IL16 | Q14005 | Pro-interleukin-16 | CVII |
| IL1RL2 | Q9HB29 | Interleukin-1 receptor-like 2 | CVII |
| IL6 | P05231 | Interleukin-6 | CVII |
| IL7R | P16871 | Interleukin-7 receptor subunit alpha | CM |
| ITGAM* | P11215 | Integrin alpha-M | CM |
| ITGB1BP2 | Q9UKP3 | Melusin | CVII |
| KIM1 | Q96D42 | Kidney Injury Molecule | CVII |
| KIT | P10721 | Mast/stem cell growth factor receptor Kit | CM |
| LCN2* | P80188 | Neutrophil gelatinase-associated lipocalin | CM |
| LEP | P41159 | Leptin | CVII |
| LILRB1 | Q8NHL6 | Leukocyte immunoglobulin-like receptor subfamily B member 1 | CM |
| LILRB2 | Q8N423 | Leukocyte immunoglobulin-like receptor subfamily B member 2 | CM |
| LILRB5 | O75023 | Leukocyte immunoglobulin-like receptor subfamily B member 5 | CM |
| FCG2B | P31994 | Low affinity immunoglobulin gamma Fc region receptor II-b | CVII |
| LOX-1 | P78380 | Lectin-like oxidized LDL receptor 1 | CVII |
| LPL | P06858 | Lipoprotein lipase | CVII |
| LTBP2* | Q14767 | Latent-transforming growth factor beta-binding protein 2 | CM |
| LYVE1 | Q9Y5Y7 | Lymphatic vessel endothelial hyaluronic acid receptor 1 | CM |
| MARCO | Q9UEW3 | Macrophage receptor MARCO | CVII |
| MBL2 | P11226 | Mannose-binding protein C | CM |
| MEGF9 | Q9H1U4 | Multiple epidermal growth factor-like domains protein 9 | CM |
| MERTK | Q12866 | Tyrosine-protein kinase Mer | CVII |
| MET | P08581 | Hepatocyte growth factor receptor | CM |
| MFAP5 | Q13361 | Microfibrillar-associated protein 5 | CM |
| MMP-12 | P39900 | Matrix metalloproteinase-12 | CVII |
| MMP-7 | P09237 | Matrix metalloproteinase-7 | CVII |
| NCAM1 | P13591 | Neural cell adhesion molecule 1 | CM |
| NEMO | Q9Y6K9 | NF-kappa-B essential modulator | CVII |
| NID1 | P14543 | Nidogen-1 | CM |
| NOTCH1 | P46531 | Neurogenic locus notch homolog protein 1 | CM |
| NRP1 | O14786 | Neuropilin-1 | CM |
| OSMR | Q99650 | Oncostatin-M-specific receptor subunit beta | CM |
| PAM | P19021 | Peptidyl-glycine alpha-amidating monooxygenase | CM |
| PAPPA | Q13219 | Pappalysin-1 | CVII |
| PAR-1 | P25116 | Proteinase-activated receptor 1 | CVII |
| PARP-1 | P09874 | Poly [ADP-ribose] polymerase 1 | CVII |
| PCOLCE | Q15113 | Procollagen C-endopeptidase enhancer 1 | CM |
| PD-L2 | Q9BQ51 | Programmed cell death 1 ligand 2 | CVII |
| PDGFB | P01127 | Platelet-derived growth factor subunit B | CVII |
| PGF | P49763 | Placenta growth factor | CVII |
| PIgR | P01833 | Polymeric immunoglobulin receptor | CVII |
| PLA2G7* | Q13093 | Platelet-activating factor acetyl hydrolase | CM |
| PLTP* | P55058 | Phospholipid transfer protein | CM |
| PLXNB2 | O15031 | Plexin-B2 | CM |
| PRCP* | P42785 | Lysosomal Pro-X carboxypeptidase | CM |
| PRELP | P51888 | Prolargin | CVII |
| PROC | P04070 | Vitamin K-dependent protein C | CM |
| BOC | Q9BWV1 | Brother of CDO | CVII |
| PRSS2 | P07478 | Trypsin-2 | CM |
| PRSS27 | Q9BQR3 | Serine protease 27 | CVII |
| PRSS8 | Q16651 | Prostasin | CVII |
| PSGL-1 | Q14242 | P-selectin glycoprotein ligand 1 | CVII |
| PTPS | Q13332 | Receptor-type tyrosine-protein phosphatase S | CM |
| PTX3 | P26022 | Pentraxin-related protein PTX3 | CVII |
| QPCT | Q16769 | Glutaminyl-peptide cyclotransferase | CM |
| RAGE | Q15109 | Receptor for advanced glycosylation end products | CVII |
| REG1A | P05451 | Lithostathine-1-alpha | CM |
| REG3A | Q06141 | Regenerating islet-derived protein 3-alpha | CM |
| REN | P00797 | Renin | CVII |
| SAA4 | P35542 | Serum amyloid A-4 protein | CM |
| SCF | P21583 | Stem cell factor | CVII |
| SELL | P14151 | L-selectin | CM |
| SERPINA12 | Q8IW75 | Serpin A12 | CVII |
| SERPINA5 | P05154 | Plasma serine protease inhibitor | CM |
| SERPINA7 | P05543 | Thyroxine-binding globulin | CM |
| SLAMF7 | Q9NQ25 | SLAM family member 7 | CVII |
| SOD1 | P00441 | Superoxide dismutase | CM |
| SOD2 | P04179 | Superoxide dismutase [Mn], mitochondrial | CVII |
| SORT1 | Q99523 | Sortilin | CVII |
| SPARCL1 | Q14515 | SPARC-like protein 1 | CM |
| SPON2 | Q9BUD6 | Spondin-2 | CVII |
| SRC | P12931 | Proto-oncogene tyrosine-protein kinase Src | CVII |
| ST6GAL1 | P15907 | Beta-galactoside alpha-2,6-sialyltransferase 1 | CM |
| STK4 | Q13043 | Serine/threonine-protein kinase 4 | CVII |
| TCN2 | P20062 | Transcobalamin-2 | CM |
| TF | P13726 | Tissue factor | CVII |
| TGFBI | Q15582 | Transforming growth factor-beta-induced protein ig-h3 | CM |
| TGFBR3 | Q03167 | Transforming growth factor beta receptor type 3 | CM |
| TGM2 | P21980 | Protein-glutamine gamma-glutamyltransferase 2 | CVII |
| THBS2 | P35442 | Thrombospondin-2 | CVII |
| THBS4 | P35443 | Thrombospondin-4 | CM |
| THPO | P40225 | Thrombopoietin | CVII |
| TIE1 | P35590 | Tyrosine-protein kinase receptor Tie-1 | CM |
| TIE2 | Q02763 | Angiopoietin-1 receptor | CVII |
| TIMD4 | Q96H15 | T-cell immunoglobulin and mucin domain-containing protein 4 | CM |
| TIMP1 | P01033 | Metalloproteinase inhibitor 1 | CM |
| TM | P07204 | Thrombomodulin | CVII |
| TNC | P24821 | Tenascin | CM |
| TNFRSF10A | O00220 | Tumour necrosis factor receptor superfamily member 10A | CVII |
| TNFRSF11A | Q9Y6Q6 | Tumour necrosis factor receptor superfamily member 11A | CVII |
| TNFRSF13B | O14836 | Tumour necrosis factor receptor superfamily member 13B | CVII |
| TNXB | P22105 | Tenascin-X | CM |
| TRAIL-R2 | O14763 | TNF-related apoptosis-inducing ligand receptor 2 | CVII |
| UMOD | P07911 | Uromodulin | CM |
| VASN | Q6EMK4 | Vasorin | CM |
| VCAM1 | P19320 | Vascular cell adhesion protein 1 | CM |
| VEGFD | O43915 | Vascular endothelial growth factor D | CVII |
| VSIG2 | Q96IQ7 | V-set and immunoglobulin domain-containing protein 2 | CVII |
| XCL1 | P47992 | Lymphotactin | CVII |
